# Exploration of novel heterofused 1,2,4-triazine derivative in colorectal cancer

**DOI:** 10.1080/14756366.2021.1879803

**Published:** 2021-01-31

**Authors:** Justyna Magdalena Hermanowicz, Anna Szymanowska, Beata Sieklucka, Robert Czarnomysy, Krystyna Pawlak, Anna Bielawska, Krzysztof Bielawski, Joanna Kalafut, Alicja Przybyszewska, Arkadiusz Surazynski, Adolfo Rivero-Muller, Mariusz Mojzych, Dariusz Pawlak

**Affiliations:** a Department of Pharmacodynamics, Medical University of Bialystok, Bialystok, Poland; b Department of Clinical Pharmacy, Medical University of Bialystok, Bialystok, Poland; c Department of Biotechnology, Medical University of Bialystok, Bialystok, Poland; d Department of Synthesis and Technology of Drugs, Medical University of Bialystok, Bialystok, Poland; e Department of Monitored Pharmacotherapy, Medical University of Bialystok, Bialystok, Poland; f Department of Biochemistry and Molecular Biology, Medical University of Lublin, Lublin, Poland; g Department of Medicinal Chemistry, Medical University of Bialystok, Bialystok, Poland; h Department of Chemistry, Siedlce University of Natural Sciences and Humanities, Siedlce, Poland; i Department of Pharmacology and Toxicology, Faculty of Medicine, University of Warmia and Mazury in Olsztyn, Olsztyn, Poland

**Keywords:** 1,2,4-Triazine derivative, apoptosis, colon cancer, zebrafish

## Abstract

Colorectal cancer (CRC) is the third leading cause of cancer-related deaths in men and in women. The impact of the new pyrazolo[4,3-*e*]tetrazolo[1,5-*b*][1,2,4]triazine sulphonamide (**MM-129**) was evaluated against human colon cancer *in vitro* and in zebrafish xenografts. Our results show that this new synthesised compound effectively inhibits cell survival in BTK-dependent mechanism. Its effectiveness is much higher at a relatively low concentration as compared with the standard chemotherapy used for CRC, i.e. 5-fluorouracil (5-FU). Flow cytometry analysis after annexin V-FITC and propidium iodide staining revealed that apoptosis was the main response of CRC cells to **MM-129** treatment. We also found that **MM-129** effectively inhibits tumour development in zebrafish embryo xenograft model, where it showed a markedly synergistic anticancer effect when used in combination with 5-FU. The above results suggest that this novel heterofused 1,2,4-triazine derivative may be a promising candidate for further evaluation as chemotherapeutic agent against CRC.

## 1. Introduction

Colorectal cancer (CRC) is the third leading cause of cancer-related deaths in men and in women. It is expected to cause about 53,200 deaths during 2020^1^. Treatments against CRC are surgery, drugs (hormonal therapy and chemotherapy), radiation, and/or immunotherapy. For patients with CRC, chemotherapy remains the most common treatment. However, it is often followed by tumour relapse and acquired chemotherapy-resistance. An effective drug for therapy and prognosis after surgery still does not exist^2–4^.

Therefore, the search for new lead structures and chemical entities for the development of new effective anticancer agents is an increasingly important task in medicinal chemistry. This trend of global research includes work on the use of 1,2,4-triazine scaffold as a source for the design of biologically relevant molecules with well-known broad biological applications^5^. Thus, the 1,2,4-triazine ring is an eminent structural motif found in plentiful natural and synthetic biologically active compounds^6^. Among the known various biological activities of 1,2,4-triazines and their related benzo- and heterofused derivatives, antitumor activity deserves special attention. Recently, a few reviews on the chemistry and the biological properties of this class of compounds have been published^7–9^.

It should be emphasised that compounds with the 1,2,4-triazine nucleus condensed with five-membered heterocycles have received considerable attention because they are bioisosteric with a purine core. Among the fused 1,2,4-triazine derivatives with one heterocycle, compounds bearing a pyrrole ring, such as pyrrolo[2,1-c][1,2,4]triazine and pyrrolo[2,1-f][1,2,4]triazine, represent the most abundant class of triazine with antitumor activity^5^. Derivatives bearing this heterocyclic system are widely described as potent kinase inhibitors. Pyrrolo[2,1-f][1,2,4] triazine scaffold, miming the adenine ring of ATP, was also employed to afford other kinase inhibitors such as Met kinase inhibitors^10^.

Another interesting and little studied in the group of 1,2,4-triazines condensed with a five-membered heterocycle is the pyrazolo[4,3-*e*][1,2,4]triazine ring system. Its derivatives are the least known in the group of condensed pyrazolotriazines and were less studied compared to pyrrolotriazines. Some synthesised derivatives of this system were evaluated for their anticancer activity against five type tumour cell lines (CEM, CEM-DNR, K-562, K-562-tax, and A549) and they showed antiproliferative activity against A549 cell line in the micromolar range^11^^,^^12^. Bernat et al. reported that pyrazolo triazine derivates show increased antiproliferative and apoptotic effects in cancerous cell lines, as compared to normal tissue^13^. Another group is pyrazolo[4,3-*e*][1,2,4]triazine sulphonamides prepared as inhibitors of carbonic anhydrase hCA IX and XII with antitumor activity^14^^,^^15^. Moreover, some previously described pyrazolotriazine sulphonamides were found to have dose-dependent antiproliferative effects against two Bcr-Abl positive cancer cell lines, K562 and BV173. Therefore, they were tested against Abl kinase^16^. On the other hand, our earlier study showed that tricyclic derivatives of the pyrazolo[4,3-*e*][1,2,4]triazines condensed with triazole or tetrazole were the most active against cancer cell lines^17^^,^^18^. Replacement of triazole with a tetrazole ring results in increase of the anticancer activity.

**Figure 1.**
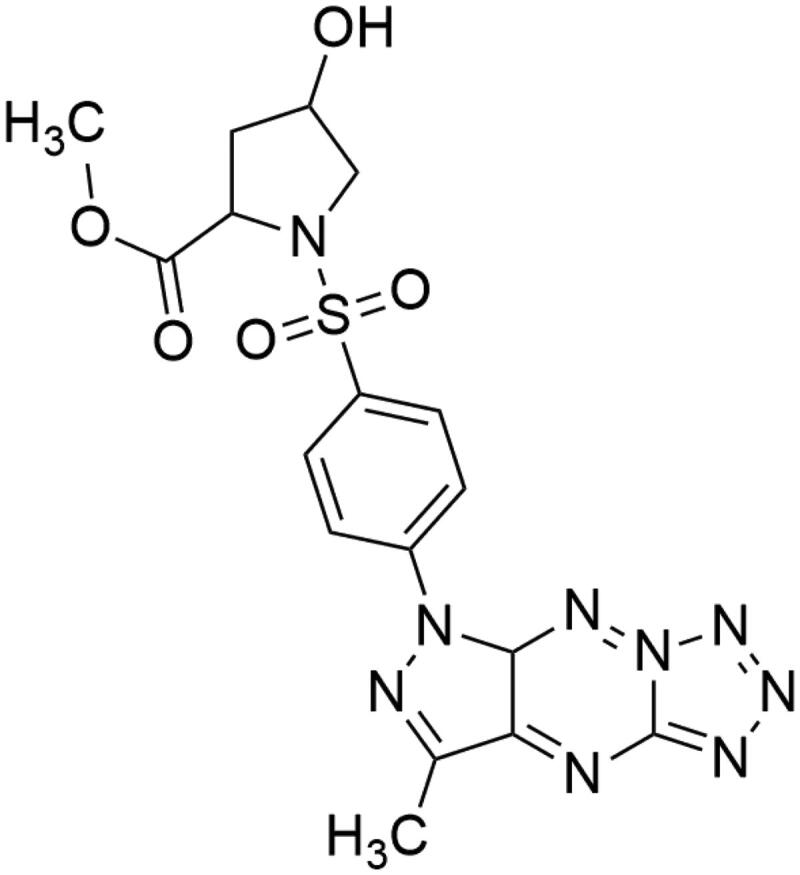
Chemical structure of **MM-129**.

In our previous study, we described synthesis and characterisation of novel pyrazolo[4,3-*e*]tetrazolo[1,5-*b*][1,2,4]triazine sulphonamides differing in the structure of the sulphonamide group^19^. Screening results revealed that compound **MM-129** presented in Figure 1 exhibited strong inhibition activity towards DLD-1 and HT-29 (two of the cell lines of CRC). It showed potent antiproliferative effects against colon cancer cell lines with IC_50_ values 3.1 µM compared to 5-fluorouracil (5-FU) and roscovitine (RSC) with values above 10 µM. **MM-129** has a similar chemical structure to RSC, which is in the clinical trial phase. We also evaluated the mechanism of action of novel sulphonamide derivative and found that it plays anticancer roles through inhibition of Bruton’s tyrosine kinase (BTK) and activation of apoptosis. In this study, we successfully developed zebrafish xenografts with human DLD-1 and HT-29 CRC cells and validated these models with anticancer drug 5-FU, used clinically to treat cancer patients. We found that **MM-129** effectively inhibits tumour development in both DLD-1 and HT-29 xenografts.

## 2. Materials and methods

### 2.1. Synthesis

#### 2.1.1. General

Melting points were determined on a Mel-Temp apparatus and were uncorrected. ^1^H and ^13^C NMR spectra were recorded on a Varian spectrometer (400 MHz for ^1^H and 100 MHz for ^13^C). The chemical shift values were expressed in ppm (part per million) with tetramethylsilane (TMS) as an internal reference. The relative integrals of peak areas agreed with those expected for the assigned structures. The molecular weight of the final compounds was assessed by electrospray ionisation mass spectrometry (ESI/MS) on Agilent Technologies 6538 UHD Accurate Mass Q-TOF LC/MS compositions (Santa Clara, CA) were within ±0.4% of the calculated values. For preparation and spectroscopic data of intermediate compounds, see the literature^19^.

#### 2.1.2. Synthesis of sulphonamide (3)

1-[(*Para*-chlorosulfonylphenyl)-3-methyl-5-methylsulfonyl]-1*H*-pyrazolo[4,3-*e*][1,2,4]triazine^19^ (194 mg, 0.5 mmol) was dissolved in anhydrous acetonitrile (5 mL) and *cis*-4-hydroxy-l-proline methyl ester hydrochloride (296 mg, 1.75 mmol) was added. Then, sodium bicarbonate (147 mg, 1.75 mmol) was added to the resulting mixture. The reaction was stirred overnight at room temperature, and then the reaction mixture was concentrated *in vacuo* to afford the crude sulphonamide, as a yellow solid. The residue was purified on silica gel using a mixture of CH_2_Cl_2_:EtOH (25:1) as eluent to give the appropriate sulphonamide as a yellow solid.

##### 2.1.2.1. Methyl 4-hydroxy-1-[4-(3-methyl-5-methylsulfonyl-1H-pyrazolo[4,3-e][1,2,4]triazin-1-yl)phenylsul-fonyl]pyrrolidine-2-carboxylate (3)

Yield 96%. Melting point: 112–114 °C; ^1^H NMR (acetone) *δ*: 2.07–2.13 (m, 1H), 2.15–2.21 (m, 1H), 2.85 (s, 3H), 3.45 (dt, 1H, *J*_1_=11.2 Hz, *J*_2_=1.7 Hz), 3.59 (s, 3H), 3.65 (dd, 1H, *J*_1_=10.8 Hz, *J*_2_=4.2 Hz), 3.73 (s, 3H), 4.08 (d, 1H, *J* = 6.8 Hz, OH exchange with D2O), 4.36 (t, 1H, *J* = 7.9 Hz), 4.42 (m, 1H), 8.16 (d, 2H, *J* = 8.7 Hz), 8.67 (d, 2H, *J* = 9.1 Hz); ^13^C NMR (acetone) *δ*: 11.26, 23.33, 40.21, 41.15, 57.60, 60.83, 70.11, 120.80, 130.32, 137.24, 142.19, 147.31, 149.60, 162.92, 173.10. HRMS (ESI, *m/z*) calcd. for C_18_H_20_N_6_O_7_S_2_ [M + H] 497.0908. Found [M + H] 497.0904. Anal. calcd. for C_18_H_20_N_6_O_7_S_2_: C, 43.54; H, 4.06; N, 16.93. Found: C, 43.60; H, 4.15; N, 16.78.

#### 2.1.3. Synthesis of tricyclic sulphonamide (MM-129)

Sulphonamide derivative (**3**) with a methylsulfonyl group (164 mg, 0.33 mmol) was dissolved in anhydrous ethanol (5 mL), and sodium azide (21 mg, 0.33 mmol) was added. The reaction mixture was refluxed until the substrate disappeared (control TLC). Then, the solvent was evaporated and the crude product was purified using column chromatography and CH_2_Cl_2_:MeOH (50:1) mixture as eluent to give the final compound as a yellow solid.

##### 2.1.3.1. Methyl 4-hydroxy-1-[4-(7-methyl-5H-pyrazolo[4,3-e]tetrazolo[1,5-b][1,2,4]triazin-5-yl)phenylsulfo-nyl]pyrrolidine-2-carboxylate (MM-129)

Yield 83%. Melting point: 122–128 °C; ^1^H NMR (acetone) *δ*: 2.04–2.18 (m, 2H), 3.39 (d, 1H, *J* = 11.2 Hz), 2.85 (s, 3H), 3.62 (d, 1H, *J* = 12 Hz), 3.77 (s, 3H), 4.33 (d, 2H, *J* = 8.3 Hz), 4.57 (bs, 1H, OH), 8.08 (d, 2H, *J* = 9.2 Hz), 8.48 (d, 2H, *J* = 9.2 Hz); ^13^C NMR (acetone) *δ*: 11.26, 41.14, 52.53, 57.59, 60.82, 70.22, 120.79, 130.32, 137.23, 142.19, 147.30, 149.60, 162.92, 173.09. HRMS (ESI, *m/z*) calcd. for C_17_H_17_N_9_O_5_S [M + H] 459.10734. Found [M + H] 460.11469. Anal. calcd. for C17H17N9O4S: C, 44.44; H, 3.73; N, 27.44. Found: C, 44.35; H, 3.89; N, 27.20.

### 2.2. Transfection

HT-29 and DLD-1 cell lines were transfected with Lipofectamine 3000 (Invitrogen, Carlsbad, CA). A day before transfection, 5 × 10^4^ cells were seeded per well in 24-well plate. Transfection was performed using 1000 ng of the plasmid DNA (pmR-mCherry Vector, Clontech, Mountain View, CA, cat no. 632542), 1.5 μL Lipofectamine^®^3000 Reagent and 1 μL P3000™ Reagent per well, following the manufacturer’s protocol. The cells were selected 48 h upon transfection for another 2 weeks in 1 mg/mL of Geneticin (G418) (Thermo Fisher, Waltham, MA). Polyclonal cells after selection were reseeded on 96-well plate in density one cell per well. A monoclonal clone with high mCherry expression was selected for further research. After selection, the monoclonal cells remained stable (mCherry expression) without the addition of G418 for 4 weeks before experiments were carried out.

### 2.3. Zebrafish handling, establishment of xenograft

Colon cancer DLD-1 and HT-29 cells were transfected before transplantation. The zebrafish embryos (wild-type AB, Experimental Medicine Center, Medical University of Lublin, Lublin, Poland) were manually dechorionated 36 h post-fertilisation (hpf), and after another 12 h were anaesthetised by placing in 0.04 mg/mL ethyl 3-aminobenzoate methanesulfonate tricaine, which is a water soluble, fast-acting anaesthetic agent. Zebrafish embryos were then transfer to a thin film of low-melting-point agarose to stabilise the fish in a lateral position. Colon cancer cells were loaded into a borosilicate glass needle pulled by a P-1000 Next Generation Micropipette Puller (Sutter Instrument Company, Novato, CA). A suspension containing about 100–200 cells was injected into the inferior section of the yolk sac in a single injection by using an electronically regulated air-pressure microinjector (Narishige IM-300 Microinjector). After injection, the zebrafish were washed once with fish water and transferred to six-well plate containing 2 mL of fresh fish water. DLD-1 and HT-29-xenografts (72 hpf) were incubated at 33 °C with **MM-129** (10 µM) and 5-FU (50 µM) and a combination of these drugs for 48 h. Zebrafish embryos develop successfully to hatching from 22 to 32 °C. The temperature experienced during early development has effects that persist into adulthood on energy metabolism pathways and acclimation capacity^20^. The temperatures higher than 28.5 °C accelerated embryonic development^21^. Although embryos are normally allowed to develop at 28.5 °C and human cells at 37 °C, a compromise at 33 °C works well.

### 2.4. Microscope imaging

Living zebrafish embryos were anaesthetised with tricaine and embedded in a lateral orientation. The animals were analysed for cytoplasmic fluorescence intensity. Images of the cells were acquired using an EVOS M5000 Imaging System with the following filters: GFP (470/22 nm excitation; 510/42 nm emission) and Texas Red (585/29 nm excitation; 624/40 nm emission). Image analysis was performed using ImageJ v1.51 software (National Institute of Health, Bethesda, MD).

Cells were plated in 96‐well culture plates optimised for imaging applications at 1 × 10^4^ cells per well. After treatment, the cells were fixed with a 3.7% formaldehyde solution at room temperature for 15 min and permeabilised with a 0.1% Triton X‐100 solution at room temperature for 10 min. Then, non‐specific binding was blocked (3% BSA). After that time, the cells were rinsed, incubated with mouse monoclonal antibodies against phospho BTK (Thermo Fisher Scientific, Waltham, MA, Cat# 44-1355G RRID:AB_2533598) for 1 h at room temperature. Then, the cells were rinsed and incubated with FITC conjugated secondary goat polyclonal antibody against mouse (Sigma-Aldrich, St. Louis, MO, Cat# F0257, RRID:AB_259378) for 60 min in the dark. After washing, the nuclei were stained with Hoechst 33342 (2 μg/mL). Cells were analysed using confocal microscope BD Pathway 855 using a ×20 objective.

### 2.5. Zebrafish drug-screening assay

The zebrafish (*Danio rerio*) were maintained at 28.5 °C in E3 buffer in 30 L aquaria at a rate of one fish per litre of water with cycles of 14/10 h of light/darkness and fed in accordance with the guidelines established by the Research Animals Department of the RSPCA. The use of animals in scientific research in Europe is governed by the Directive 2010/63/EU of 22 September 2010. According to EU Directive earliest life-stages of zebrafish (embryo and eleutheroembryo cultures) are regarded as equivalent to *in vitro* cell culture therefore, do not fall into the regulatory frameworks dealing with animal experiments. In contrast, experiments with the free‐feeding larvae older than 120 h of development are classified as animal experiments and require permission. The rationale is that active hunting reflects a perception of environmental stimuli that go beyond simple reflexes. This is taken as an indication of a mature nervous system that controls behaviour. In our experiment, we used zebrafish larvae younger than 120 hpf (hours post-fertilisation) therefore ethic approval was not required. Zebrafish embryos were obtained from mating adults, maintained and raised as described previously^22^^,^^23^. Zygote period cleaving eggs were transferred to six-well plastic cell culture plates filled with embryo medium E3. The eggs (10–12 per well) were exposed to RSC (50 µM), 5-FU (50 µM), and **MM-129** (10 µM) for 3 h^24^^,^^25^. The final volume of the medium in each well was 2 mL. DMSO was used as a drug solvent. The final concentration of DMSO in the wells did not exceed the damaging concentration of above 0.1%. The mock control embryos were incubated in embryo medium in the presence of 0.1% DMSO. The drug effect was recognised when all the eggs from one well changed in the same characteristic manner. Each experiment was carried out in three independent experiments. Observations of cell division and development of the zebrafish eggs were carried out using SteREO Discovery.V8 stereo microscope (Zeiss, Jena, Germany) once every 15 min within the first three hours of incubation.

### 2.6. Cell culture

Human colorectal adenocarcinoma cell line DLD-1 (CCL-221) and HT-29 (HTB-38) was purchased from the American Type Culture Collection (ATCC, Manassas, VA). The DLD-1 line histologically is the most similar to a primary tumour. Line HT-29, in turn, is used to assess multidrug resistance, absorption of nutrients, and chemically induced differentiation of enterocytes. Cells (passage number range of 6–8) were cultured in RPMI 1640 medium (Sigma, St. Louis, MO) and McCoy’s 5a medium (ATCC), respectively, complemented with 10% of foetal bovine serum (FBS) and 1% of antibiotics: penicillin/streptomycin. The cells were maintained in an incubator, which provides optimal growth conditions for cell culture: 5% CO_2_, 37 °C, and humidity in a range of 90–95%. The cells were cultured in 100 mm plates (Sarstedt, Newton, NC). Subsequently, after obtaining a subconfluent cell culture, the cells were detached with 0.05% trypsin and 0.02% EDTA phosphate buffered saline without calcium and magnesium (Corning, Corning, NY). Then, utilising a haemocytometer, the number of cells was quantified and seeded at a density of 5 × 10^5^ cells per well in six-well plates (“Nunc”) in 2 mL of the growth medium (RPMI 1640 and McCoy’s 5a). In the present study, cells that obtained 80% of confluency were used.

### 2.7. Cell viability assay

Cytotoxicity of newly synthesised derivative was estimated by MTT assay as we described in an earlier article^26^. Briefly, DLD-1 and HT-29 cells were seeded in six-well plates “Nunc” at a density of 5 × 10^5^ cells/well and incubated for 24 h in optimal growth conditions. Subsequently, **MM-129**, and reference drugs, RSC, 5-FU, at concentrations 1, 3, and 10 µM were added in duplicate and the plates were incubated for another 24 h. Next, the plates were washed with PBS three times. Then, 1 mL PBS and 50 µL of 5 mg/cm^3^ MTT solution were added and the incubation was continued for 4 h. MTT tetrazolium is converted in viable cells into purple crystals of formazan. The conversion does not occur in dead cells. After the required time, the supernatant was removed and formazan crystals were dissolved in DMSO. The absorbance was measured at a wavelength 570 nm. The absorbance result obtained in the control was taken as 100% and the viability of the cells incubated with the tested compounds was showed as a percentage of the control cells. The values from the samples were obtained from three independent experiments done in duplicate (*N* = 6).

### 2.8. [^3^H]thymidine incorporation assay

DLD-1 and HT-29 cells were cultured in six-well plates and treated with various concentrations (1 µM, 3 µM, and 10 µM) of the tested compound and reference drugs, RSC and 5-FU, for 24 h. Following the incubation, the cells were treated with 0.5 µCi of radioactive [^3^H]thymidine (specific activity 6.7 Ci/mmol) for 4 h in optimal growth condition. Afterward, the medium was removed and cells were washed two times with 1 mL of 0.05 M Tris–HCl buffer containing 0.11 M NaCl. In the next step, the cells were washed twice with 1 mL of 5% TCA acid and then dissolved in 1 mL of 0.1 M NaOH containing 1% SDS. Following 5 min of incubation, the obtained cell lysates were transferred to scintillation vials previously filled with 2 mL scintillation fluid. The radioactivity was quantified on Scintillation Counter (Packard Tri-Carb Liquid Scintillation Counter 1900 TR, Perkin Elmer, Inc., Waltham, MA). [^3^H]thymidine uptake was expressed as dpm/well. The intensity of DNA biosynthesis in the control was taken as 100%. The values from the samples were obtained from three independent experiments done in duplicate (*N* = 6) and presented as a percentage of the control cells.

### 2.9. Western blot

DLD-1 and HT-29 cells were incubated (24 h) with **MM-129**, RSC, and 5-FU in two concentrations 1 µM and 3 µM. Western blotting was performed using a standard method described previously^27^. The nitrocellulose was incubated with rabbit polyclonal Tyr223 antibody against phospho BTK (Cell Signaling Technology, Boston, MA, Cat# 5082P, RRID:AB_10557114). Samples used for electrophoresis consisted of 20 µg of protein from six pooled cell extracts (*N* = 6).

### 2.10. Flow cytometry assessment of Annexin V binding

Induction of apoptosis was examined using Apoptosis Detection Kit II analysed using a flow cytometry, as previously described^28^. During programmed cell death (PCD), the phosphatidylserine (PS) is transferred from the inner cell membrane to the cell surface. DLD-1 and HT-29 cells were incubated (24 h) with **MM-129** and with the reference drugs: RSC and 5-FU. All compounds were used in two concentrations: 1 µM and 3.0 µM. Following incubation, the cells were dyed with FITC-labelled annexin V and propidium iodide. It allows to identify viable, necrotic, early, and late apoptotic cells. The positive controls were cells in which apoptosis was induced by the addition of 2 μL of 3% formaldehyde. The cells were placed in a refrigerator for 15 min to induce apoptosis. Three controls were made: the first contained control cells and propidium iodide; the second, control cells and annexin V-FITC; and the third, control cells and propidium iodide and annexin V-FITC. Cells cultured in medium without the tested compounds were used as the negative control. The values were obtained from three independent experiments (*n* = 3). The experiment was performed using the BD FACSCanto II flow cytometer and the results were parsed with FACSDiva software (BD Biosciences Systems, San Jose, CA).

### 2.11. Analysis of mitochondrial membrane potential

Changes in the mitochondrial membrane potential (MMP) were provided with flow cytometry using JC-1 MitoScreen kit (BD Biosciences, San Jose, CA). In normal cells, JC-1 (1,1′,3,3′-tetraethyl-5,5′,6,6′-tetrachloroimidacarbocyanine iodide) aggregates in the mitochondrial matrix. Nevertheless, in apoptotic and necrotic cells, this lipophilic dye diffuses out of mitochondria and stains cells with a green fluorescent. DLD-1 and HT-29 colon cancer cell lines covering about 80% of the plate were incubated with the novel synthesised compound **MM-129**, and the reference drugs, RSC and 5-FU, for 24 h in an incubator at 37 °C, 5% CO_2_. All compounds were used at 1 µM and 3.0 µM concentrations. Following incubation, the medium was removed and the cells were washed two times with the required buffer. Then, the cells were suspended in a 10 µg/mL JC-1 dye and incubated in the dark for 15 min. The cells were washed with PBS and analysed using BD FacsCanto II flow cytometer. The results were assessed using BD FacsDiva software (BD Biosciences Systems, San Jose, CA). The values were obtained from three independent experiments (*n* = 3).

### 2.12. Caspase activity assays

Detection of caspase-8, -9, -10, and -3/7 activity was assessed with the appropriate kit (caspase-8: FLICA Caspase-8 Assay Kit, caspase-9: FLICA Caspase-9 Assay Kit, caspase-10: FLICA Caspase-10 Assay Kit, caspase-3/7: FLICA Caspase-3/7 Assay Kit). DLD-1 and HT-29 were incubated for 24 h with **MM-129** and reference drugs, RSC and 5-FU, in two concentrations: 1 µM and 3 µM. Following incubation, the cells were washed twice with cold PBS. Subsequently, the cells were resuspended in the required buffer (93 µL was gently mixed with 5 µL required FLICA and 2 µL Hoechst 33342) and incubated at 37 °C for 60 min. Then, the cells were washed twice with apoptosis wash buffer and centrifuged at 300×*g*. After preparing, the samples, the cells were resuspended in 100 µL buffer and labelled with 10 µg/mL propidium iodide. The experiment was performed using the BD FACSCanto II flow cytometer and the results were parsed with FACSDiva software (BD Biosciences Systems, San Jose, CA). The values were obtained from three independent experiments done in duplicate (*N* = 6).

### 2.13. Statistical analysis

Shapiro–Wilk’s test of normality was used for data distribution analysis. The normally distributed data were expressed as mean ± SD. Multiple group comparisons were performed by one-way analysis of variance (ANOVA), and significant differences between the groups were assessed using the Tukey–Kramer test. Calculations were performed using GraphPad 6 Prism software (La Jolla, CA). The differences were deemed statistically significant when *p* < 0.05. Measurement of fluorescence intensity was analysed using ImageJ 1.50a software (National Institute of Health, Bethesda, MD).

## 3. Results

### 3.1. Synthesis of novel 1,2,4-triazine derivative

**Scheme 1.**
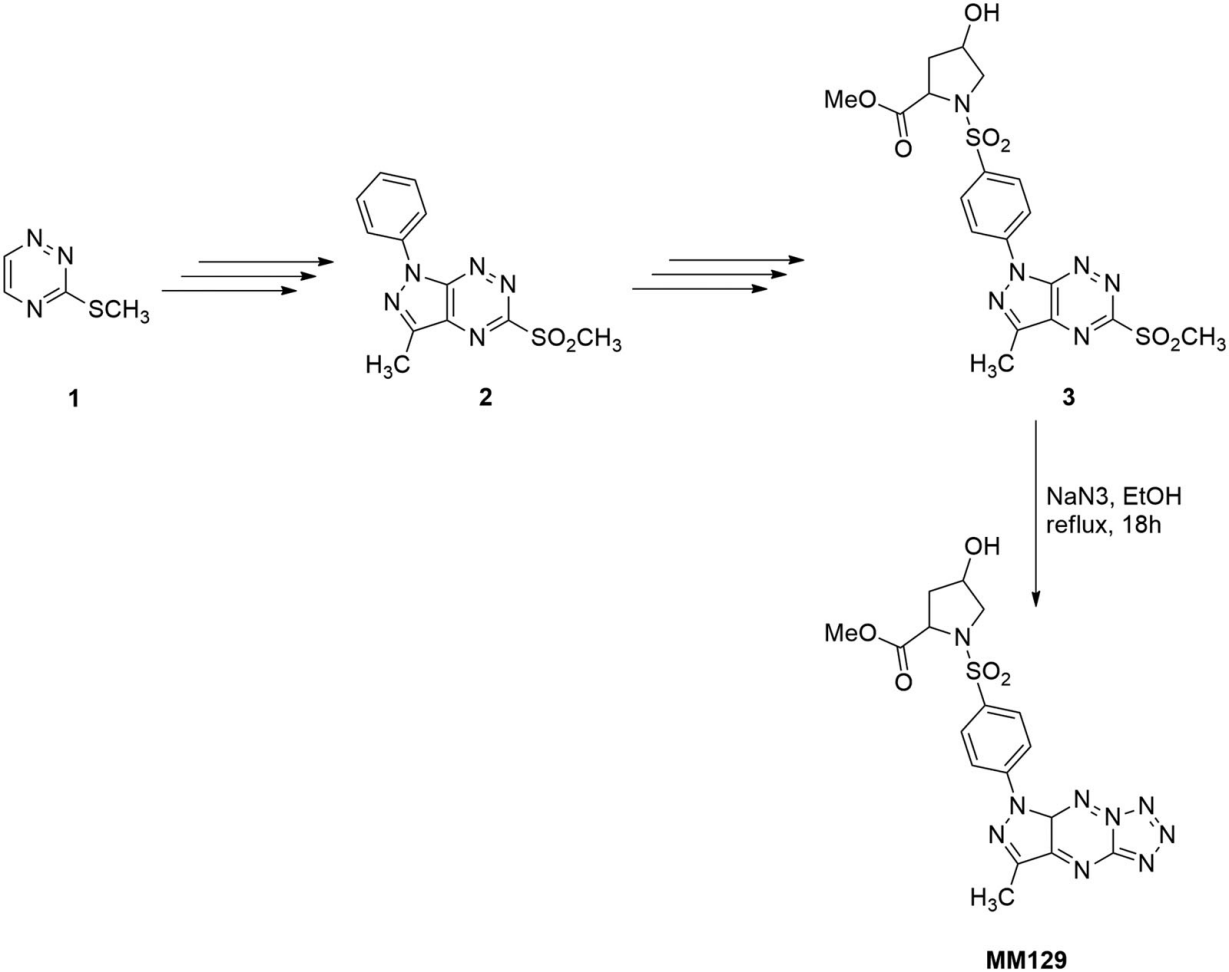
Synthetic pathway for production of new sulphonamide **MM-129**.

The target compound **MM-129** was obtained by a multi-step synthesis, starting from 1,2,4-triazine (1), according to the methods described in the literature (Scheme 1)^16^^,^^19^^,^^29^. Briefly, the synthesis of 3-methyl-5-methylsulfonyl-1-phenyl-1*H*-pyrazolo[4,3-*e*][1,2,4]triazine (2) was performed based on the reaction of nucleophilic substitution of hydrogen in 1,2,4-triazine and oxidation process under two-phase-transfer catalysis conditions^15^. The final new tricyclic sulphonamide **MM-129** was achieved by a convenient multiple procedure consisting of the following steps: chlorosulfonylation, reaction with l-proline derivative, and nucleophilic substitution reaction of methylsulfonyl group with sodium azide^19^. The structures and the purity of the newly synthesised compound **MM-129** were characterised using the ^1^H and ^13^C NMR, and HRMS methods together with elemental analysis.

### 3.2. MM-129 inhibits tumour development in zebrafish xenografts

**Figure 2.**
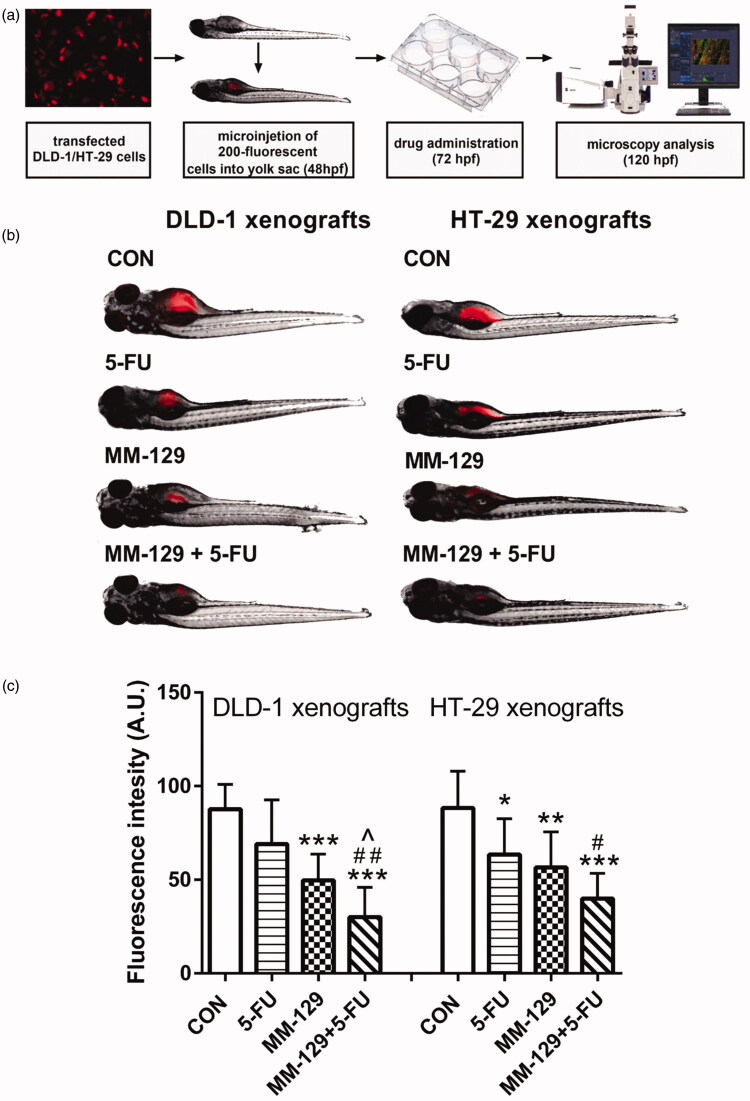
Schematic of xenograft assay and analysis of tumour development (a). Site-specific injection (yolk sac) of transfected (red) colon cancer cells (DLD-1 and HT-29) into 48 hpf zebrafish embryos and imaging analysis of tumour growth after 48 h of incubation with **MM-129** (50 µM), 5-FU (50 µM), or a combination of these agents (b). Quantification of total fluorescence by colon cancer cells three days after injection (c) *n* = 8, *p < .05, **p < .01, ***<.001 vs. CON; ^*p* < .05 vs. **MM-129**, ^#^*p* < .05, ^##^*p* < .01 vs. 5-FU. Data were presented as mean ± standard deviation (SD), and analysed using one-way analysis of variance (ANOVA). *p* < .05 was considered statistically significant.

DLD-1 and HT-29 cells stably transfected with a plasmid encoding mCherry fluorescent protein (pmR-mCherry) were implanted into the yolk sac of wild-type (WT) zebrafish 48 hpf embryos. The scheme of the experimental protocol is presented in Figure 2(a). Three days after cell injection, solid tumours were established in 100% of DLD-1 (*N* = 32) and HT-29 xenografts (*N* = 32). The embryos were then incubated with **MM-129**, 5-FU, or a combination of these compounds for 48 h. The control group received the **MM-129** solvent (0.1% DMSO/PBS). In both DLD-1 and HT-29 xenografts, we observed a significant reduction in tumour development in the **MM-129** treated group compared with the control group, as reflected by the drop in fluorescence intensity (49.73 ± 13.93 vs. 87.71 ± 13.22 and 56.62 ± 18.91 vs. 88.26 ± 19.69, both *p* < 0.01) (Figure 2(b,c)). Incubation with **MM-129**+5-FU significantly intensified the reduction in the number of cancer cells in both DLD-1 (30.12 ± 15.83) and HT-29 (40.0 ± 13.42) xenografts compared with the appropriate control (87.71 ± 13.22 and 88.26 ± 19.69; both *p* < 0.001). The combination of **MM-129**+5-FU showed more pronounced anticancer activity than 5-FU alone in DLD-1 and HT-29 cells (69.06 ± 23.67 and 63.46 ± 19.1, *p* < 0.01 and *p* < 0.05; respectively). Similarly, the combination of these compounds was also more effective in reducing cancer cells than **MM-129** alone in DLD-1 (49.73 ± 13.93; ^*p* < 0.05). These results indicate that **MM-129** showed a markedly synergistic anticancer effect when used in combination with 5-FU.

### 3.3. Antiproliferative activity of MM-129 in zebrafish model

**Figure 3.**
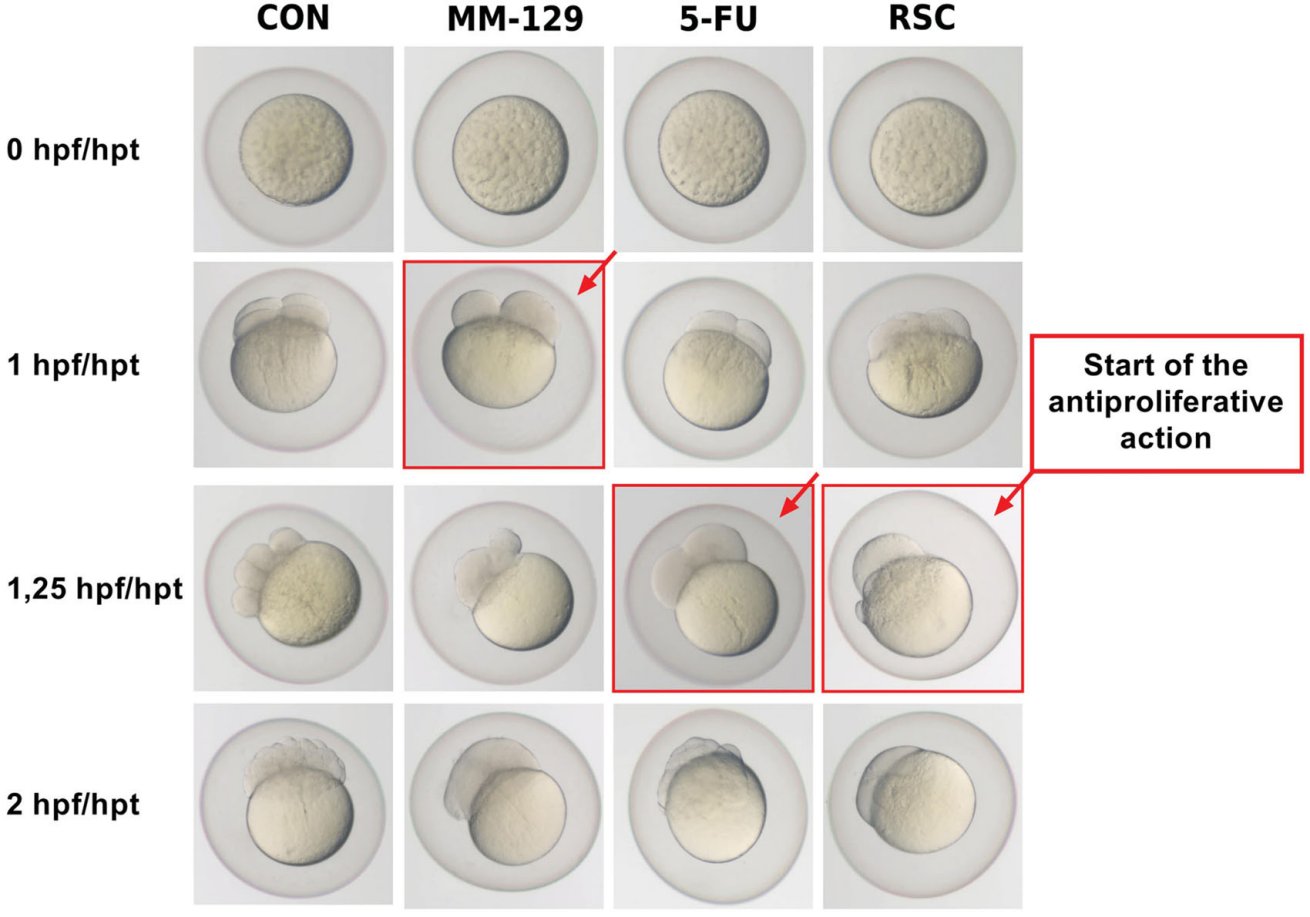
Effect of **MM-129**, 5-fluorouracil (5-FU), and roscovitine (RSC) on cell division in the zebrafish embryo. Zebrafish eggs after 0, 1, 1.25, and 2 h of exposure to **MM-129**, 5-FU and RSC; *n* = 10. hpf: hours post fertilisation; hpt: hours post treatment.

The antiproliferative activity of **MM-129** and the reference drugs (RSC, 5-FU) were also examined in a zebrafish embryo model (Figure 3). At a constant temperature of 28.5 °C (ST), the ZF egg cleaves first at 45 min post fertilisation forming the two-cell stage egg. Zebrafish embryos reach 4-cell, 8-cell, and 64-cell stages within 1, 1.25, and 2 h post fertilisation (hpf), respectively. Subsequently, they enter into the blastula stage (2.25–5.25 hpf), the gastrula stage (5.25–10 hpf), and the segmentation stage (10–24 hpf) to finally hatch out between 48 and 72 hpf^30^^,^^31^. In untreated eggs, we observed normal embryo development revealed in consecutive synchronous cleavages. This process was disturbed in embryos exposed to **MM-129**, RSC, and 5-FU. At 1 hpt (hours post treatment), **MM-129**-treated eggs showed a deterioration of cell division, cell disorientation, and initial signs of cell fusion. Within the next 30 min, fusion dramatically progressed in **MM-129**-treated eggs, while the control eggs continued cell division without any apparent delay. When zebrafish eggs were treated with RSC and 5-FU, they developed normally for 1 h up to the four-cell stage, and then at the eight-cell stage they showed proliferation arrest. After two hours of incubation, we noticed complete cell fusion and lysis in groups exposed to **MM-129** and RSC. 5-FU did not show such strong changes during the study period. No alteration in cell division and development in untreated control eggs were observed.

### 3.4. Biological activity of novel 1,2,4-triazine derivative

**Figure 4.**
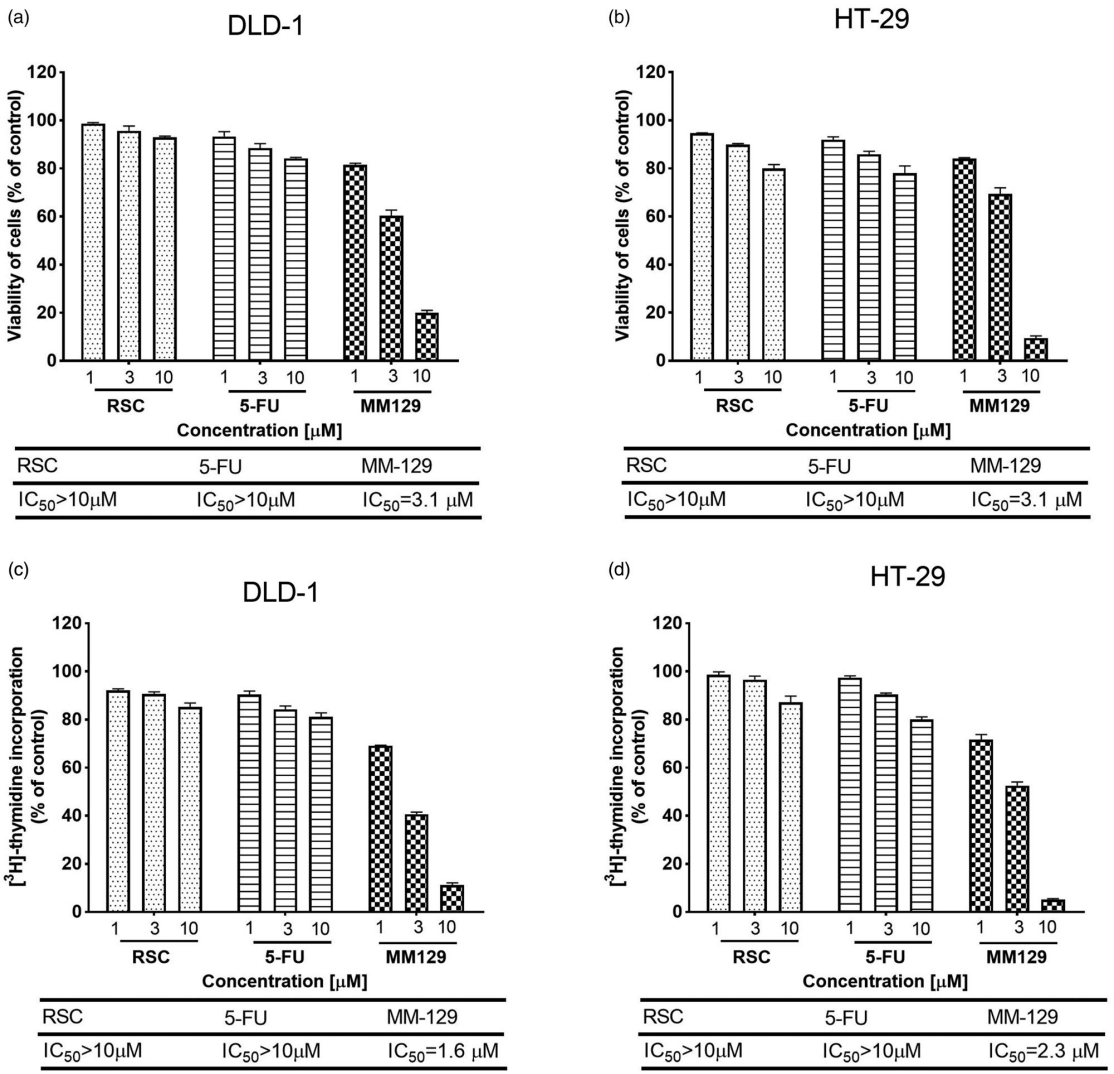
Viability of DLD-1 (a) and HT-29 (b) colon cancer cells treated for 24 h with different concentrations of **MM-129**, roscovitine (RSC) and 5-fluorouracil (5-FU), and the antiproliferative effects of the studied compounds in DLD-1 (c) and HT-29 (d) colon cancer cells measured by the inhibition of [^3^H]thymidine incorporation into DNA. Mean values ± SD from three independent experiments (*n* = 3) done in duplicate are presented.

The effect of pyrazolo[4,3-*e*]tetrazolo[1,5-*b*][1,2,4]triazine derivative on the viability of colon adenocarcinoma cell lines DLD-1 and HT-29 was assessed with MTT assay. The study showed that the tested compound has a much higher cytotoxic potency than the reference drugs in both cell lines. In DLD-1 and HT-29 cells, the IC_50_ value was 3.1 µM for **MM-129**, while for RSC and 5-FU, it was above 10 µM (Figure 4(a,b)). To confirm the antiproliferative effects of the new synthesised compound, its effect on DNA biosynthesis in DLD-1 and HT-29 colon adenocarcinoma cells was investigated. The estimation was made by measuring the incorporation of radioactive labelled thymidine into the DNA of the cancer cells after 24-h incubation with various concentrations of the tested and reference compounds (1 µM, 3 µM, and 10 µM). The results are presented in Figure 4(c,d). **MM-129** was able to inhibit the growth of human colon adenocarcinoma cell lines in a concentration dependent manner. The concentration of **MM-129** needed to inhibit the incorporation of [^3^H]thymidine into DNA in DLD-1 cells by 50% (IC_50_) was 1.6 µM, whilst in HT-29 was 2.3 µM. This effect was stronger than that of RSC and 5-FU in both cell lines.

### 3.5. MM-129 inhibits intracellular signalling pathways via the down-regulation of BTK expression

**Figure 5.**
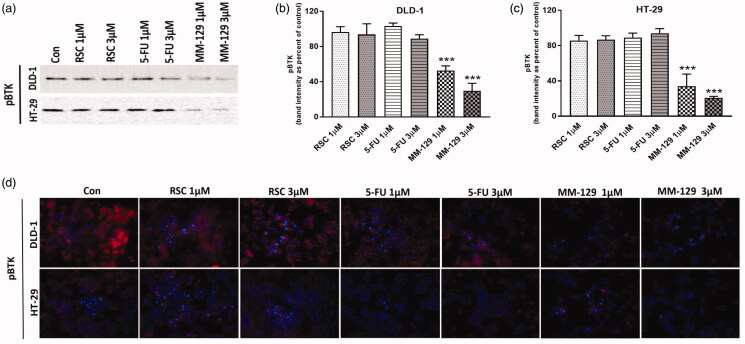
The down-regulation of Bruton’s kinase (BTK) expression by **MM-129**. Phosphorylated BTK (pBTK) expression was determined by Western blot (a) and confocal microscopy (d) in DLD-1 and HT-29 cells treated with **MM-129**, roscovitine and 5-flurouracil in two concentrations: 1 µM and 3 µM for 24 h. The band staining was quantified by densitometry (b, c). Samples used for electrophoresis consisted of 20 µg of protein from six pooled cell extracts (*n* = 6). Data were presented as mean ± standard deviation (SD), and analysed using one-way analysis of variance (ANOVA). ***p<.001 vs. CON intensity of cytoplasmic/nuclear fluorescence was analysed in cell populations. Images of cells labelled with FITC were obtained using a 515LP emission laser and a 488/10 excitation laser (d).

Bruton’s tyrosine kinase is a non-receptor tyrosine kinase involved in the activation of signalling pathways responsible for cell maturation and viability. The incubation of DLD-1 and HT-29 cells with **MM-129** for 24 h resulted in downregulation of phosphorylated BTK (pBTK) expression compared with control as well as compared with RSC and 5-FU in both cell lines. The strongest reduction in BTK level was exhibited by the **MM-129** at a concentration of 3 µM (Figure 5(a–c)). These results were confirmed by confocal microscopy, where we also observed a significant decrease in BTK expression after **MM-129** stimulation (Figure 5(d)).

### 3.6. MM-129 induces apoptosis through phosphatidylserine externalisation and loss of mitochondrial membrane potential

**Figure 6.**
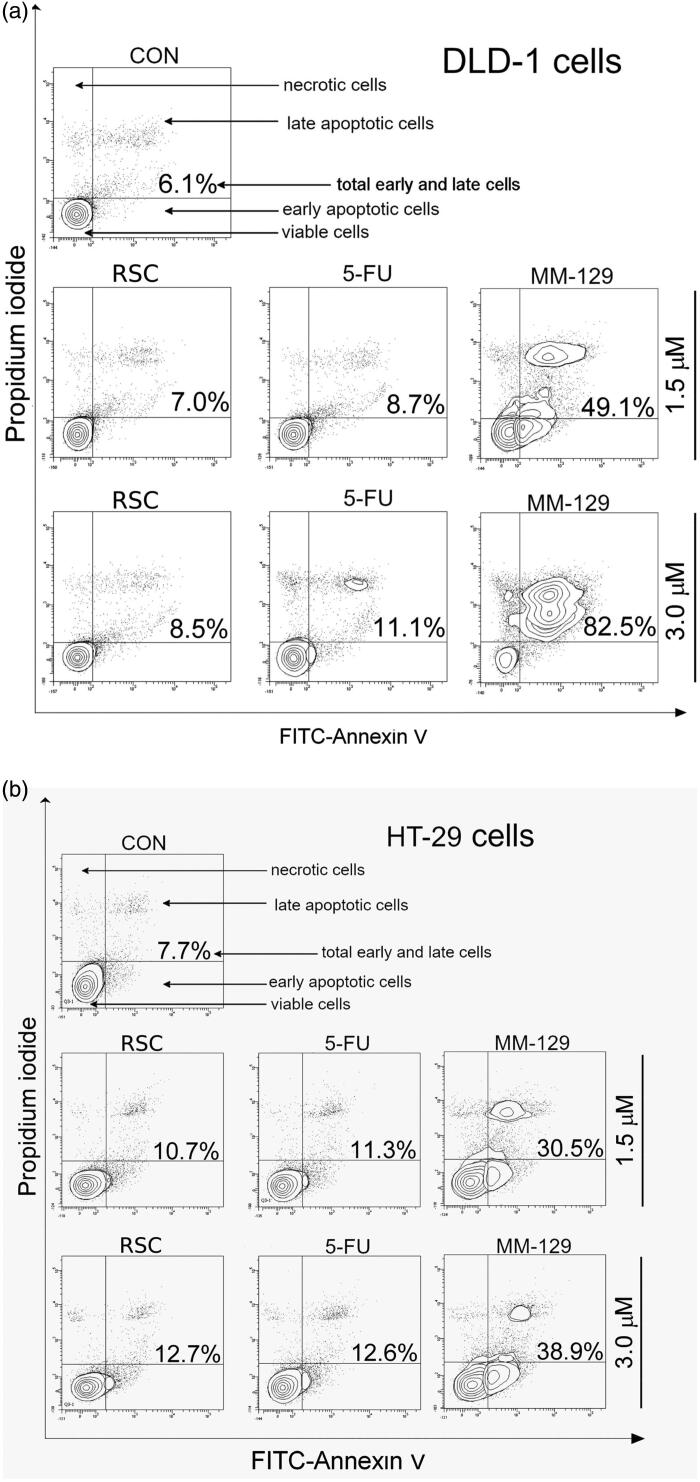
Representative flow cytometry dot-plots for annexin V‐FITC-propidium iodide assay of DLD-1 (a) and HT-29 (b) colon cancer cells after 24 h of incubation with roscovitine (RSC), 5-fluorouracil (5-FU), or **MM-129** (1 μM and 3 μM).

Determination of the apoptosis status of DLD-1 and HT-29 cells after 24 h of incubation with 1,2,4-triazine derivative, RSC, and 5-FU was assessed by dual annexin V and propidium iodide staining using flow cytometry. One of the characteristic early changes in the cells during PCD is displacement of phosphatidylserine from the internal to the external cell membrane–externalisation of phosphatidylserine. Dual staining allows to detect live cells (not binding annexin V and PI), early-apoptotic cells (binding annexin V and not binding PI), late-apoptotic cells (binding annexin V and PI), and necrotic cells (binding PI and not binding annexin V). Following 24 h incubation with the studied compounds, the activation of PCD in DLD-1 and HT-29 cell lines was observed (Supplementary Figure 1Sa, 1Sb). A total of 6.1% of apoptotic and 1.9% necrotic cells were observed in the population of DLD-1 control cells (Figure 6(a)). The strongest proapoptotic properties on this cell line were exhibited by the **MM-129** compound at a concentration of 3 µM, where the percentage of early and late apoptotic cells was about 82.5%. In the HT-29, the highest number of apoptotic cells–38.9% was also seen in response to analogous **MM-129** concentration vs. 12.7%, 12.6%, and 7.7% in cells treated with RSC, 5-FU or controls, respectively (Figure 6(b)). These data reveal that the cytotoxic activity of pyrazolo[4,3-*e*]tetrazolo[1,5-*b*][1,2,4]triazine derivatives against DLD-1 and HT-29 cancer cells is due to the induction of PCD.

**Figure 7.**
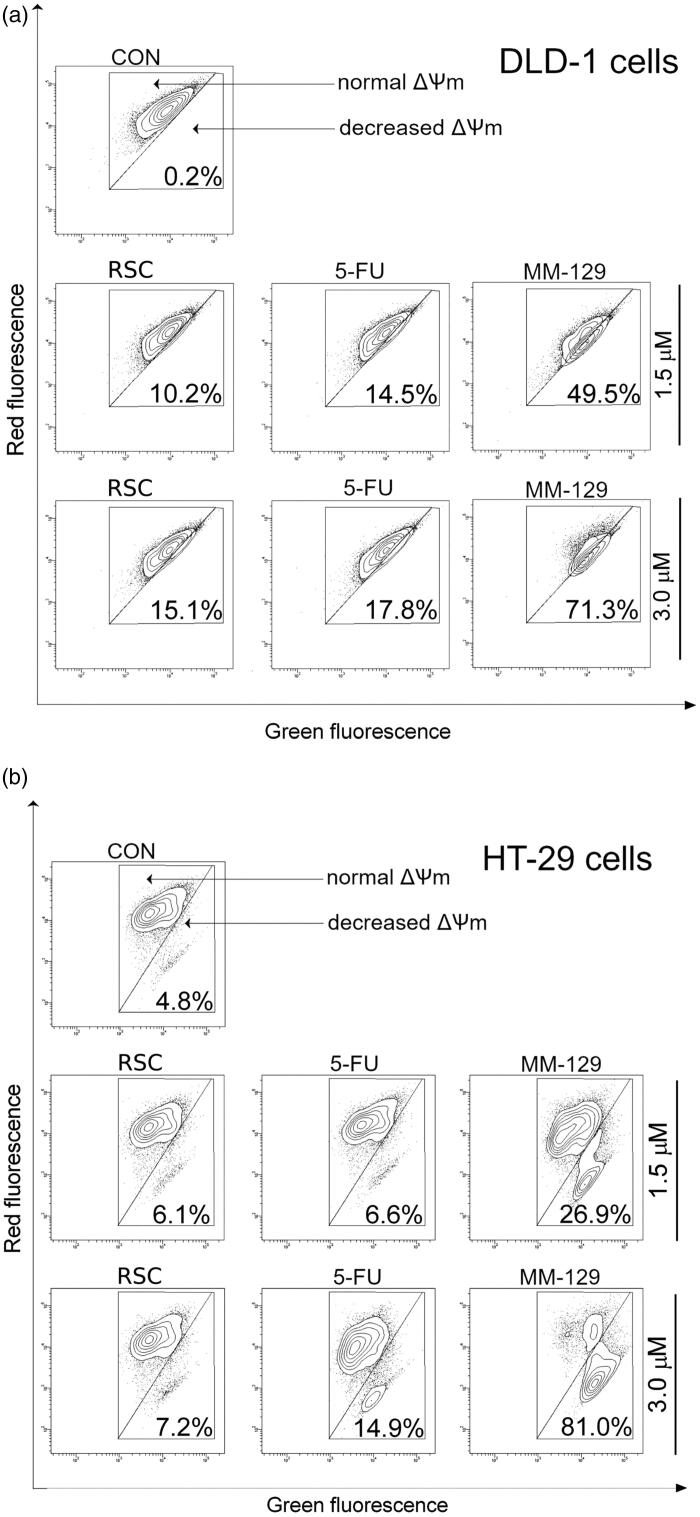
Representative dot-plots presenting the loss of mitochondrial membrane potential (ΔΨm) of DLD-1 (a) and HT-29 (b) colon cancer cells treated for 24 h with roscovitine (RSC), 5-fluorouracil (5-FU), and **MM-129** (1 μM and 3 μM).

A decrease in MMP is one of the earliest changes associated with PCD. During apoptosis, the permeability of the external and the internal mitochondrial membrane is increased. As a consequence, the mitochondrial proteins are released into the cytosol through the outer membrane, e.g. cytochrome c and apoptosis inducing factor (AIF). The exposure to RSC, 5-FU, and **MM-129** for 24 h resulted in the loss of membrane integrity in both colon adenocarcinoma cell lines (Supplementary Figure 1Sc, 1Sd). The rate of MMP-disrupted cells was increased in a concentration dependent manner. This effect was particularly seen in the case of **MM-129** at a concentration of 3 µM, where the percentage of cells with decreased levels of MMP reached 71.3% and 81% for DLD-1 (Figure 7(a)) and HT-29 (Figure 7(b)), respectively. Moreover, in the case of **MM-129**, a clear concentration dependent effect was observed. This suggests that pyrazolo[4,3-*e*]tetrazolo[1,5-*b*][1,2,4]triazine-induced MMP disruption involved in the intrinsic apoptosis pathway.

### 3.7. MM-129 increases caspases activity

**Figure 8.**
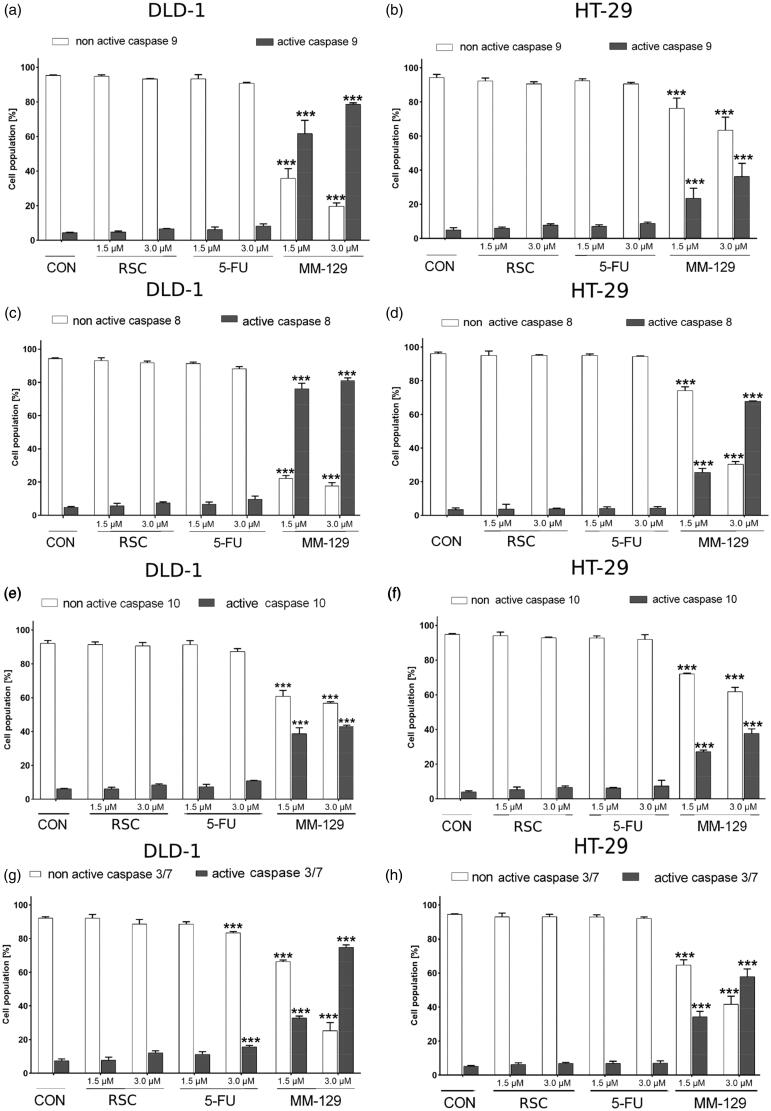
Flow cytometric analysis of caspase-9, caspase-8, caspase-10, and caspases-3/7 activation in the populations of DLD-1 (a, c, e, g) and HT-29 (b, d, f, h) colon cancer cells treated for 24 h with roscovitine (RSC), 5-fluorouracil (5-FU), and **MM-129** (1 μM and 3 μM). Mean percentage values from three independent experiments done in duplicate (*N* = 6) were presented as mean ± standard deviation (SD), and analysed using one-way analysis of variance (ANOVA). ***p<.001 vs. CON.

Caspases play a major role in the activation of apoptosis. There are two main pathways that can lead to the activation of caspases: the internal (mitochondrial) and the external (receptor) pathway. The first path leads to mitochondrial membrane perturbation and release of cytochrome C to the cytosol. This protein binds to the Apaf-1 and procaspase-9 in the cytoplasm creating a multiprotein complex-apoptosome, which activates executive caspases. Hence, the effect of RSC and 5-FU or **MM-129** on the activation of caspase-9 was examined after 24 h incubation (Supplementary Figure 2Sa, 2Sb). The results revealed that the novel pyrazolo[4,3-*e*]tetrazolo[1,5-*b*][1,2,4]triazine derivative leds to an increased expression of caspase-9 in both cell lines. In the DLD-1 cell culture, compound **MM-129** at a 3 µM concentration showed a stronger effect on the activation of caspase-9 than the reference drugs. In this case, the percentage of cells with the active form of this initiator caspase was 78.7% (Figure 8(a,b)). In the HT-29 cell line, 36.4% of cells had an activated initiation of caspase-9 after 24 h incubation with **MM-129** at the same concentration.

Stimulation of the extrinsic pathway starts with the activation of death receptors. After binding the appropriate death ligand, the receptors accumulate in clusters in the cell membrane and promote the recruitment of adapter proteins. These proteins have the death effector domain (DED), through which they interact with procaspase-8 and procaspase-10. The receptor, adapter protein, and procaspase form the complex DISC, that led to the activation of caspase-8 and caspase-10, and subsequently cell death. The activation of caspase-8 and caspase-10 was determined after 24-h treatment with RSC, 5-FU, and **MM-129** by FLICA Caspase-8 Assay Kit (Supplementary Figure 3Sa, 3Sb) and FLICA Caspase-10 Assay Kit (Supplementary Figure 4Sa, 4Sb). It was shown that tested compound increased the expression of the active form of caspase-8 and caspase-10 in both cell lines. The highest percent of cells with the active form of caspase 8 was observed for **MM-129** at a 3 µM concentration, and it was 81.2% on DLD-1 cell line and 67.8% on HT-29 cell (Figure 8(c,d)). Similar effect on the activation of caspase-10 in DLD-1 was also exhibited by **MM-129** at a 3 µM concentration; the percentage of cells with the active form of initiator caspase-10 was 42.9%. The highest percentage of HT-29 cells expressing active caspase-10 was also evoked by compound **MM-129**, and it was 37.9% (Figure 8(e,f)).

The caspases-3/7 belong to the group of executive caspases. Procaspases-3/7 are cleaved into their active forms, caspase-3 and caspase-7, by activated caspases-8, -10, and -9. Using flow cytometry, it was examined whether the process of PCD in the studied cell lines initiated by new pyrazolo[4,3-*e*]tetrazolo[1,5-*b*][1,2,4]triazine derivative and reference compounds is related with caspase-3/7. The obtained results showed that **MM-129** increased the expression of the active form of caspases-3/7 in DLD-1 and HT-29 cell lines (Supplementary Figure 5Sa, 5Sb). The strongest effect was observed in cells incubated in the presence of **MM-129** at a 3 µM concentration in both DLD-1 and HT-29. The percentage of cells with active executive caspase-3/7 was 74.8% on DLD-1 and 57.9% on HT-29 cells, respectively (Figure 8(g,h)).

## 4. Discussion

Roscovitine, (2*R*)-2-{[6-(benzylamino)-9-isopropyl-9*H*-purin-2-yl]amino}-1-butanol also called CY-202 or seliciclib, is a low molecular weight purine derivative with a characteristic ring structure. It belongs to cyclin-dependent kinase (CDK) protein inhibitors, which play a key role in regulating the cell cycle, promoting its progression or transition between the individual phases^32^^,^^33^. Roscovitine has been shown to possess anticancer activity in various *in vitro* and *in vivo* models^34^^,^^35^. The antitumor action of this chemotherapeutic has been tested using cancer xenografts of LoVo human CRC cells, MESSA-DX5 human uterine carcinoma cells, or MDA-MB 231 human breast cancer cells grafted into CD1 nude mice^36^^,^^37^.

The aim of the present study was to evaluate the anticancer potential of new pyrazolo[4,3-*e*]tetrazolo[1,5-*b*][1,2,4]triazine sulphonamide (**MM-129**), which has a similar chemical structure to RSC and may be found applicable in the treatment of patients with colorectal carcinoma. Undoubtedly, this kind of cancer is a significant problem, both from the medical and social point of view. For this purpose, preclinical studies were conducted on new 1,2,4-triazine derivative using cell lines and a zebrafish cancer model.

The anticancer action of **MM-129** was investigated in a zebrafish embryo xenograft model. The xenograft experiment using DLD-1 and HT-29 human CRC cells grafted into the zebrafish yolk showed a significant antitumor effect of **MM-129** with a 56% (DLD-1 xenografts) and 64% (HT-29 xenografts) reduction in the number of cancer cells compared with the control group. A basic element of colon cancer treatment is surgery, which is aimed at obtaining intestinal tissue free of cancer. Chemotherapy complements surgical intervention and is based mainly on 5-FU. For chemo, either the FOLFOX (5-FU, leucovorin, and oxaliplatin) or CapeOx (capecitabine and oxaliplatin) regimens are used most often, but some patients may get 5-FU with leucovorin or capecitabine alone based on their age and health needs (NCCN Guidelines 2018). It should be emphasised that **MM-129** turned out to be more potent in inhibiting tumour development compared with 5-FU in both DLD-1 and HT-29 xenografts. Furthermore, **MM-129** clearly showed synergistic anticancer effects when used in combination with the latter.

An antiproliferative and antitumor activity of new triazine derivative was also confirmed in the zebrafish embryo model. Genetic similarities with humans cause that zebrafish are often used as a strong and cost-effective animal model for cancer research and the discovery of cancer drugs. The small size, transparency of zebrafish embryos, easy manipulation, short testing period, and small amount of tested drugs are undoubtedly important aspects giving zebrafish and advantage over the mouse/rat model of cancer. A preliminary screening test showed that **MM-129** possesses the most biological activity; therefore, we decided to use it for further studies. This compound blocked cell division in a zebrafish embryo model at the four-cell stage of embryonic, development and these disorders were noticeable earlier than in the case of RSC or 5-FU. Two hours of incubation led to complete cell fusion and lysis in groups exposed to **MM-129**. Cell division blockade and developmental arrest caused by this compound were irreversible.

We also tested the synthesised compound for its effects on cell survival using DLD-1 and HT-29 cell lines. Cytotoxicity assay revealed that the novel **MM-129** was more effective than hitherto used agents, including RSC and reference drug 5-FU. The available evidence indicates dose-dependent cytotoxicity of RSC with IC_50_ ranging from 15 to 25 μM in multiple myeloma cells, from 18 to 22 µM in cervical carcinoma (C33A, HCE-1, HeLa, SiHa) and 67.55 µM in human glioblastoma A172^38–40^. Similar data exist for 5-FU, which induces significant CRC cell growth inhibition at a much higher concentration of 50 µM^41^. New 1,2,4-triazine derivative displayed a significantly higher potency compared with the currently used compounds as well as other triazine derivatives. 1,2,4-Triazine derivatives bearing thiophene moieties were tested for their cytotoxic effect against Hep-G2, MCF-7, and HCT-116. Their impact on the viability of cells after 48 h incubation were assessed using paclitaxel as a reference drug and they presented IC_50_ values in the range of 8.79–36.41 µM^42^. Other 1,2,4-triazine derivatives were screened by the NCI for their *in vitro* antitumor activity. The strongest compound showed antiproliferative activity against the 60 NCI human tumour cell lines, with growth inhibition GI_50_ ranging from 1.47 to 12.2 µM, more potent than 5-FU used as a reference drug^5^. In our study, the IC_50_ values for RSC and 5-FU were at least threefold higher than **MM-129** (3.1 µM).

**Figure 9.**
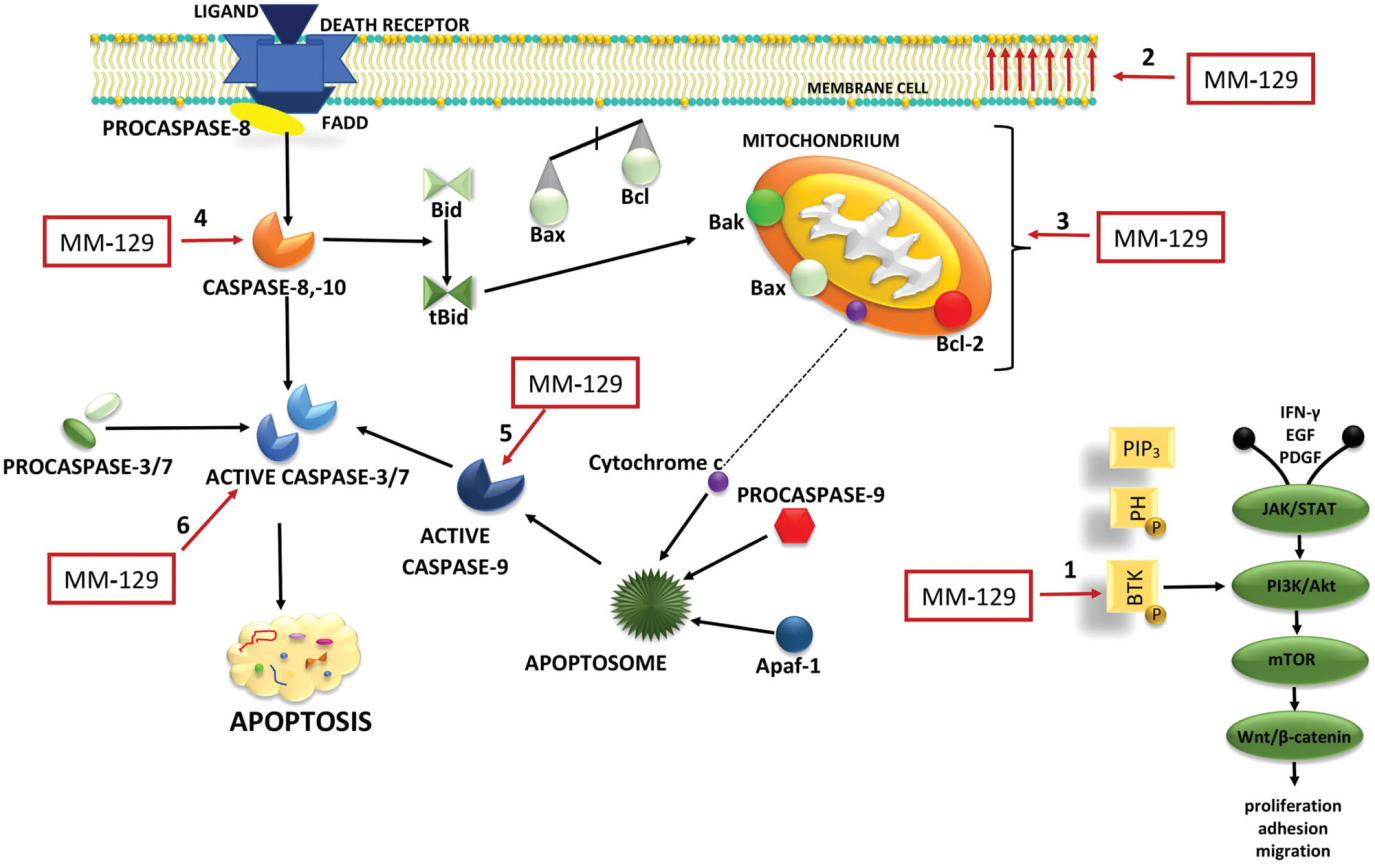
Schematic representation of possible anticancer mechanisms of **MM-129**. 1: BTK inhibition; 2: phosphatidylserine (PS) externalisation; 3: loss of mitochondrial membrane potential; 4: activation of extrinsic pathway of apoptosis; 5: the activation of internal (mitochondrial) apoptosis; 6: activation of executive caspases. The schematic illustration was created in Adobe Photoshop and Photophea software. Akt: protein kinase B; Apaf-1: apoptotic protease activating factor 1; β-catenin: protein responsible for transduction; Bak: Bcl-2 homologous antagonist/killer; Bax: Bcl-2-associated X protein; Bcl-2: antiapoptotic protein; Bid: Bax-like BH3 protein; tBid: truncated BID; BTK: Bruton’s tyrosine kinase; EGF: epidermal growth factor; FADD: Fas-associated death domain protein; IFNγ: interferon gamma; JAK2: non-receptor tyrosine kinase; mTOR: mammalian target of rapamycin; PDGF: platelet-derived growth factor; PI3K: phosphoinositide 3-kinases; PIP3: phosphatidylinositol-3,4,5-triphosphate; PH: pleckstrin homology domain; STAT: signal transducer and activator of transcription; Wnt: family of secreted lipid-modified signalling glycoproteins.

An increasing number of experimental and clinical studies indicates a major role of BTK not only in B cell malignancies but also in other solid tumours, including breast, ovarian, prostate, and colon cancer (BJP)^43–47^. Furthermore overexpression of this kinase in colon cancer has previously been observed^47^. To investigate the mechanism involved in antitumor effects of **MM-129** in DLD-1 and HT-29 cells, the level of pBTK was determined. We observed that new 1,2,4-triazine derivative significant decreased levels of active form of BTK in both cell lines. Previously, it has been demonstrated that BTK plays an important role in the development of tumours via activation of antiapoptotic pathways^48^. In turn BTK inhibition besides blocking NFκB-DNA binding, reducing proliferation, survival and cell migration, is able to induce apoptosis^49^. This is in line with our results because treatment of DLD-1 and HT-29 with **MM-129** resulted in BTK inhibition and activation of apoptosis (Figure 9).

Programmed cell death is a crucial process in animal development and tissue homeostasis, responsible for the elimination of senescent, damaged, and unhealthy cells from the body^50^. The mechanism of apoptosis is highly complex, multistage, and involves many pathways. Based on the mechanism of RSC action, which is widely described as an effective inducer of apoptosis and apoptosis-dependent cell death (Hep-G2, MCF-7, HCT-116), we assessed the impact of new 1,2,4-triazine derivative on several apoptotic pathways^40^^,^^42^^,^^51^. As has been summarised in Figure 9, the tested compound affected several cellular targets involved in the induction of apoptosis-dependent cell death. First, we noticed significant accumulation of apoptotic colon cancer cells with externalised PS after incubation with **MM-129**. To explore the cellular mechanism, by which the synthesised compound triggers induction of apoptosis, we examined the alterations of the mitochondrial transmembrane potential and caspase activity by using flow cytometry analysis. We found that new derivative increased the number of cells with decreased levels of MMP and contributed to a marked increase in the activity of all tested caspases compared with the control cells in both lines.

Our results are in agreement with Jiang et al., who synthesised 3-amino-1,2,4-benzotriazine-1,4-dioxide derivatives and demonstrated that they determined apoptosis in cancer cells by inducing a loss of MMP^52^. Mechanism studies of other 1,2,4-triazine derivatives bearing an extra 1,2,4-benzotriazine-1-oxide chromophore also revealed that the cytotoxic activity seems to be due to the activation of caspase 3/7 and G2/M arrest-mediated apoptosis^53^. Herein, RSC or 5-FU used at low concentrations had no effect on apoptosis. This is in line with previous reports indicating that 5 FU induces caspase-dependent apoptosis of CRC cells at concentrations of 50 µM^41^. Roscovitine also exhibited a clear proapoptotic effect in the A172 and G28 only at 50 µM and 100 µM, respectively^40^. We intentionally used the same concentrations for all compounds, both tested and reference. Our results clearly indicate that new 1,2,4-triazine derivative is an apoptosis-inducing factor with higher efficiency than RSC or 5-FU.

## 5. Conclusions

New 1,2,4-triazine derivative **MM-129** has been shown to exert potent antitumor activity on CRC. It effectively inhibits cell survival in BTK-dependent mechanism and apoptosis was the main response of CRC cells to **MM-129** treatment. This new synthesised compound successfully reduces tumour development in zebrafish embryo xenograft model. **MM-129** possesses cytotoxic, antiproliferative, and proapoptotic activity and its effectiveness is much higher compared with the standard chemotherapy for CRC, i.e. 5-FU. The discovery of an innovative therapeutic option that improves the prospects of treating patients is currently a crucial challenge. There is a real chance that the results presented in this study will help in the development of a new, effective therapy, which could be an attractive alternative to the already existing methods of colon cancer treatment.

## Supplementary Material

Supplemental Material

## References

[1] 2020. Available from: https://www.cancer.org/cancer/colon-rectal-cancer/about/key-statistics.html [last accessed 14 Feb 2020].

[2] Hu C, Bailey CE, You YN, et al. Time trend analysis of primary tumor resection for stage IV colorectal cancer: less surgery, improved survival. JAMA Surg 2015;150:245–51. 25588105 10.1001/jamasurg.2014.2253

[3] Forster S, Radpour R. Molecular immunotherapy: promising approach to treat metastatic colorectal cancer by targeting resistant cancer cells or cancer stem cells. Front Oncol 2020;10:569017. 33240813 10.3389/fonc.2020.569017 PMC7680905

[4] Lizardo DY, Kuang C, Hao S, et al. Immunotherapy efficacy on mismatch repair-deficient colorectal cancer: from bench to bedside. Biochim Biophys Acta Rev Cancer 2020;1874:188447. 33035640 10.1016/j.bbcan.2020.188447 PMC7886024

[5] Cascioferro S, Parrino B, Spanò V, et al. An overview on the recent developments of 1,2,4-triazine derivatives as anticancer compounds. Eur J Med Chem 2017;142:328–75. 28851503 10.1016/j.ejmech.2017.08.009

[6] Yurttas L, Ciftci GA, Temel HE, et al. Biological activity evaluation of novel 1,2,4-triazine derivatives containing thiazole/benzothiazole rings. Anticancer Agents Med Chem 2017;17:1846–53. 28356019 10.2174/1871520617666170327151031

[7] Kumar R, Sirohi TS, Singh H, et al. 1,2,4-Triazine analogs as novel class of therapeutic agents. Mini Rev Med Chem 2014;14:168–207. 24479860 10.2174/1389557514666140131111837

[8] Rao DS, Kumar GVP, Pooja B, et al. An extensive review on 1,2,3 and 1,2,4-triazines scaffold-valuable lead molecules with potent and diverse pharmacological activities. Der Chem Sin 2016;7:101–30.

[9] Arshad M, Khan TA, Khan MA, et al. 1,2,4-Triazine derivatives: synthesis and biological applications. Int J Pharm Sci Res 2014;5:149–62.

[10] Borzilleri R, Chen Z, Hunt JT, et al. Pyrrolotriazine kinase inhibitors, US7173031 B2; 2007.

[11] Gucký T, Frysová I, Slouka J, et al. Cyclocondensation reaction of heterocyclic carbonyl compounds. Part XIII: synthesis and cytotoxic activity of some 3,7-diaryl-5-(3,4,5-trimethoxyphenyl)pyrazolo[4,3-e][1,2,4]triazines. Eur J Med Chem 2009;44:891–900. 18632190 10.1016/j.ejmech.2008.05.026

[12] Gucký T, Řezníčková E, Džubák P, et al. Synthesis and anticancer activity of some 1,5-diaryl-3-(3,4,5-trihydroxyphenyl)-1H-pyrazolo[4,3-e][1,2,4]Triazines. Monatsh Chem 2010;141:709–14.

[13] Bernat Z, Szymanowska A, Kciuk M, et al. Review of the synthesis and anticancer properties of pyrazolo[4,3-e][1,2,4]triazine derivatives. Molecules 2020;25:3948. 10.3390/molecules25173948 PMC7504782 32872493

[14] Mojzych M, Bielawska A, Bielawski K, et al. Pyrazolo[4,3-e][1,2,4]triazine sulfonamides as carbonic anhydrase inhibitors with antitumor activity. Bioorg Med Chem 2014;22:2643–7. 24713308 10.1016/j.bmc.2014.03.029

[15] Mojzych M, Ceruso M, Bielawska A, et al. New pyrazolo[4,3-e][1,2,4]triazine sulfonamides as carbonic anhydrase inhibitors. Bioorg Med Chem 2015;23:3674–80. 25921266 10.1016/j.bmc.2015.04.011

[16] Mojzych M, Šubertová V, Bielawska A, et al. Synthesis and kinase inhibitory activity of new sulfonamide derivatives of pyrazolo[4,3-e][1,2,4]triazines. Eur J Med Chem 2014;78:217–24. 24681986 10.1016/j.ejmech.2014.03.054

[17] Mojzych M. Cytotoxic activity of some pyrazolo[4,3-e][1,2,4]triazine against human cancer cell lines. J Chem Soc Pak 2011;33:123–8.

[18] Mojzych M, Tarasiuk P, Karczmarzyk Z, et al. Synthesis, structure and antiproliferative activity of new pyrazolo[4,3-e]triazolo[4,5-b][1,2,4]triazine derivatives. Med Chem 2018;14:53–9. 29065838 10.2174/1573406413666171020114924

[19] Gornowicz A, Szymanowska A, Mojzych M, et al. The effect of novel 7-methyl-5-phenyl-pyrazolo[4,3-e]tetrazolo[4,5-b][1,2,4]triazine sulfonamide derivatives on apoptosis and autophagy in DLD-1 and HT-29 colon cancer cells. Int J Mol Sci 2020;21:5221. 10.3390/ijms21155221 PMC7432848 32717981

[20] Scott GR, Johnston IA. Temperature during embryonic development has persistent effects on thermal acclimation capacity in zebrafish. Proc Natl Acad Sci USA 2012;109:14247–52. 22891320 10.1073/pnas.1205012109 PMC3435178

[21] Pype C, Verbueken E, Saad MA, et al. Incubation at 32.5 °C and above causes malformations in the zebrafish embryo. Reprod Toxicol 2015;56:56–63. 26005098 10.1016/j.reprotox.2015.05.006

[22] Westerfield M. Embryonic and larval culture. In: The zebrafish book. A guide for the laboratory use of zebrafish (*Danio rerio*). Vol. 3. 4th ed. Eugene: University of Oregon Press; 2000:154–96.

[23] Strähle U, Scholz S, Geisler R, et al. Zebrafish embryos as an alternative to animal experiments-a commentary on the definition of the onset of protected life stages in animal welfare regulations. Reprod Toxicol 2012;33:128–32. 21726626 10.1016/j.reprotox.2011.06.121

[24] Liu NA, Jiang H, Ben-Shlomo A, et al. Targeting zebrafish and murine pituitary corticotroph tumors with a cyclin-dependent kinase (CDK) inhibitor. Proc Natl Acad Sci USA 2011;108:8414–9. 21536883 10.1073/pnas.1018091108 PMC3100964

[25] Xu Z, Hu C, Chen S, et al. Apatinib enhances chemosensitivity of gastric cancer to paclitaxel and 5-fluorouracil. Cancer Manag Res 2019;11:4905–15. 31213909 10.2147/CMAR.S196372 PMC6549793

[26] Tankiewicz-Kwedlo A, Hermanowicz JM, Surazynski A, et al. Erythropoietin enhances the cytotoxic effect of hydrogen peroxide on colon cancer cells. Curr Pharm Biotechnol 2017;18:127–37. 27903235 10.2174/1389201018666161116092907

[27] Tankiewicz-Kwedlo A, Hermanowicz JM, Domaniewski T, et al. Simultaneous use of erythropoietin and LFM-A13 as a new therapeutic approach for colorectal cancer. Br J Pharmacol 2018;175:743–62. 29160911 10.1111/bph.14099 PMC5811618

[28] Tankiewicz-Kwedlo A, Hermanowicz JM, Pawlak K, et al. Erythropoietin intensifies the proapoptotic activity of LFM-A13 in cells and in a mouse model of colorectal cancer. Int J Mol Sci 2018;19:1262. 10.3390/ijms19041262 PMC5979332 29690619

[29] Mojzych M, Rykowski A. Synthesis of functionalized 1H-pyrazolo[4,3-e][1,2,4]triazines and their fused derivatives via Ipso-substitution of methylsulfonyl group with O-, N-, S- and C-nucleophiles. Heterocycles 2004;63:1829–38.

[30] Uckun FM, Dibirdik I, Qazi S, et al. Anti-breast cancer activity of LFM-A13, a potent inhibitor of Polo-like kinase (PLK). Bioorg Med Chem 2007;15:800–14. 17098432 10.1016/j.bmc.2006.10.050

[31] Bozkurt Y. The role of PSR in Zebrafish (*Danio rerio*) at early embryonic development. In: Yusuf Bozkurt, ed. Recent advances in Zebrafish researches. IntechOpen; 2018:179–88.

[32] Dorand RD, Nthale J, Myers JT, et al. Cdk5 disruption attenuates tumor PD-L1 expression and promotes antitumor immunity. Science 2016;353:399–403. 27463676 10.1126/science.aae0477 PMC5051664

[33] Ding L, Cao J, Lin W, et al. The roles of cyclin-dependent kinases in cell-cycle progression and therapeutic strategies in human breast cancer. Int J Mol Sci 2020;21:1960. 10.3390/ijms21061960 PMC7139603 32183020

[34] Aldoss IT, Tashi T, Ganti AK. Seliciclib in malignancies. Expert Opin Investig Drugs 2009;18:1957–65. 10.1517/13543780903418445 19938906

[35] Guzi T. CYC-202 Cyclacel. Curr Opin Investig Drugs 2004;5:1311–8. 15648953

[36] McClue SJ, Blake D, Clarke R, et al. *In vitro* and *in vivo* antitumor properties of the cyclin dependent kinase inhibitor CYC202 (R-roscovitine). Int J Cancer 2002;102:463–8. 12432547 10.1002/ijc.10738

[37] Maggiorella L, Deutsch E, Frascogna V, et al. Enhancement of radiation response by roscovitine in human breast carcinoma *in vitro* and *in vivo*. Cancer Res 2003;63:2513–7. 12750274

[38] Raje N, Kumar S, Hideshima T, et al. Seliciclib (CYC202 or R-roscovitine), a small-molecule cyclin-dependent kinase inhibitor, mediates activity via down-regulation of Mcl-1 in multiple myeloma. Blood 2005;106:1042–7. 15827128 10.1182/blood-2005-01-0320 PMC1895150

[39] Cui C, Wang Y, Wang Y, et al. Exploring the relationship between the inhibition selectivity and the apoptosis of roscovitine-treated cancer cells. J Anal Methods Chem 2013;2013:389390. 23691435 10.1155/2013/389390 PMC3649549

[40] Kolodziej M, Goetz C, Di Fazio P, et al. Roscovitine has anti-proliferative and pro-apoptotic effects on glioblastoma cell lines: a pilot study. Oncol Rep 2015;34:1549–56. 26151768 10.3892/or.2015.4105

[41] Mhaidat NM, Bouklihacene M, Thorne RF. 5-Fluorouracil-induced apoptosis in colorectal cancer cells is caspase-9-dependent and mediated by activation of protein kinase C-δ. Oncol Lett 2014;8:699–704. 25013487 10.3892/ol.2014.2211 PMC4081407

[42] Saad HA, Youssef MM, Mosselhi MA. Microwave assisted synthesis of some new fused 1,2,4-triazines bearing thiophene moieties with expected pharmacological activity. Molecules 2011;16:4937–57. 21677606 10.3390/molecules16064937 PMC6264204

[43] Hendriks RW, Yuvaraj S, Kil LP. Targeting Bruton’s tyrosine kinase in B cell malignancies. Nat Rev Cancer 2014;14:219–32. 24658273 10.1038/nrc3702

[44] Eifert C, Wang X, Kokabee L, et al. A novel isoform of the B cell tyrosine kinase BTK protects breast cancer cells from apoptosis. Genes Chromosomes Cancer 2013;52:961–75. 23913792 10.1002/gcc.22091 PMC5006942

[45] Zucha MA, Wu AT, Lee WH, et al. Bruton’s tyrosine kinase (Btk) inhibitor ibrutinib suppresses stem-like traits in ovarian cancer. Oncotarget 2015;6:13255–68. 26036311 10.18632/oncotarget.3658 PMC4537012

[46] Guo W, Liu R, Bhardwaj G, et al. Targeting Btk/Etk of prostate cancer cells by a novel dual inhibitor. Cell Death Dis 2014;5:e1409. 25188519 10.1038/cddis.2014.343 PMC4540187

[47] Grassilli E, Pisano F, Cialdella A, et al. A novel oncogenic BTK isoform is overexpressed in colon cancers and required for RAS-mediated transformation. Oncogene 2016;35:4368–78. 26804170 10.1038/onc.2015.504 PMC4994017

[48] Uckun FM, Zheng Y, Cetkovic-Cvrlje M, et al. In vivo pharmacokinetic features, toxicity profile, and chemosensitizing activity of alpha-cyano-beta-hydroxy-beta- methyl-N-(2,5-dibromophenyl)propenamide (LFM-A13), a novel antileukemic agent targeting Bruton’s tyrosine kinase. Clin Cancer Res 2002;8:1224–33. 12006542

[49] Cheng S, Ma J, Guo A, et al. BTK inhibition targets in vivo CLL proliferation through its effects on B-cell receptor signaling activity. Leukemia 2014;28:649–57. 24270740 10.1038/leu.2013.358

[50] Fuchs Y, Steller H. Programmed cell death in animal development and disease [published correction appears in Cell. 2011 Dec 23;147(7):1640]. Cell 2011;147:742–58. 22078876 10.1016/j.cell.2011.10.033 PMC4511103

[51] Tirado OM, Mateo-Lozano S, Notario V. Roscovitine is an effective inducer of apoptosis of Ewing’s sarcoma family tumor cells *in vitro* and *in vivo*. Cancer Res 2005;65:9320–7. 16230394 10.1158/0008-5472.CAN-05-1276

[52] Jiang F, Yang B, Fan L, et al. Synthesis and hypoxic-cytotoxic activity of some 3-amino-1,2,4-benzotriazine-1,4-dioxide derivatives. Bioorg Med Chem Lett 2006;16:4209–13. 16777409 10.1016/j.bmcl.2006.05.095

[53] Lee CI, Huang CM, Huang WH, et al. Synthesis, preferentially hypoxic apoptosis and anti-angiogenic activity of 3-amino-1,2,4-benzotriazine-1,4-dioxide bearing alkyl linkers with a 3-amino-1,2,4-benzotriazine-1-oxide moiety. Anticancer Agents Med Chem 2014;14:1428–46. 25312508 10.2174/1871520614666141014130554 PMC4428392

