# Supplementary material for: Exploration of novel heterofused 1,2,4-triazine derivative in colorectal cancer
Source: J Enzyme Inhib Med Chem. 2021 Jan 31;36(1):535–48. doi: 10.1080/14756366.2021.1879803 (PMC7850456; doi:10.1080/14756366.2021.1879803)
Supplement: Supplemental Material [file IENZ_A_1879803_SM9052.pdf]

Figure 1S

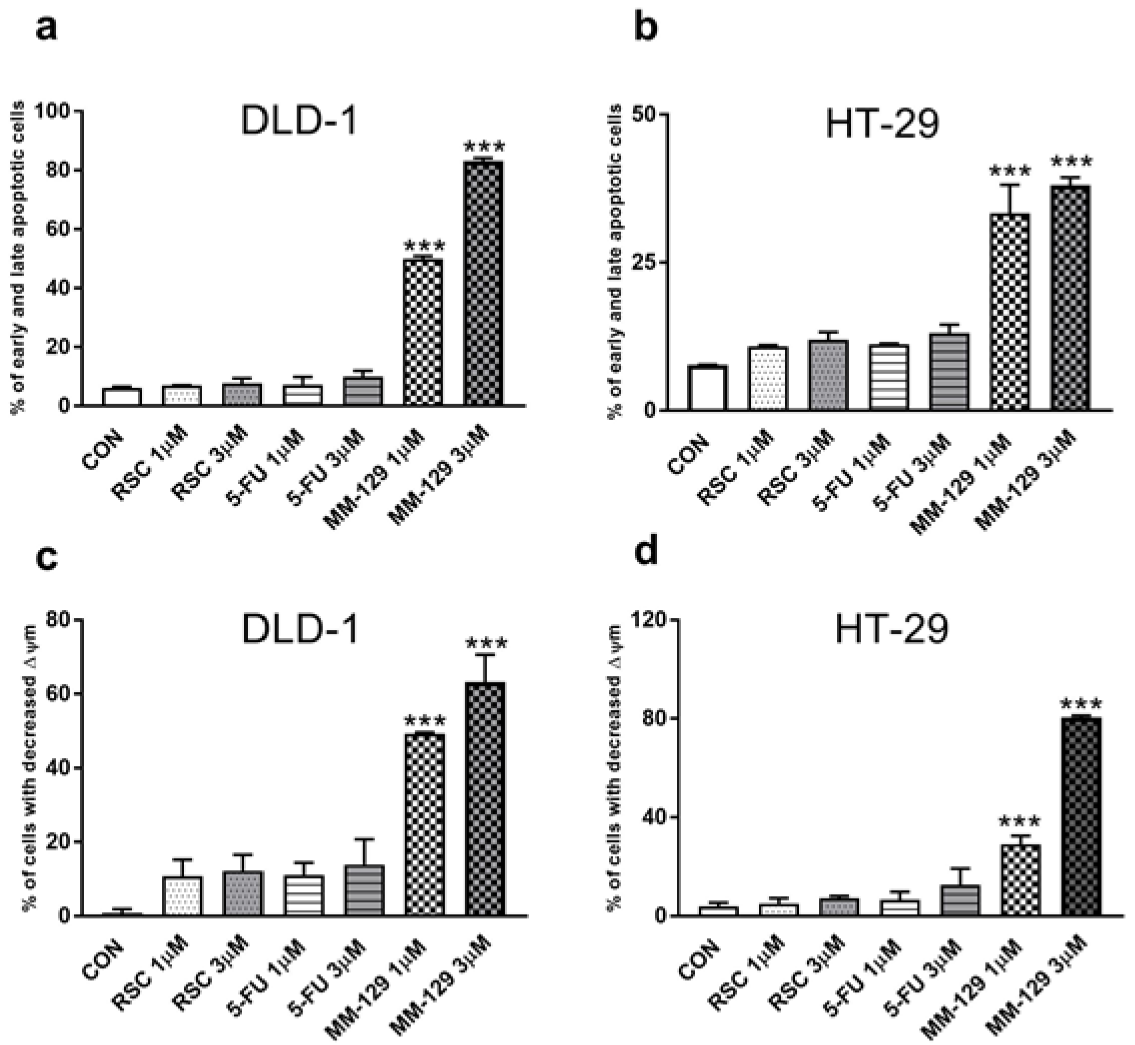

a
DLD-1
% of early and late apoptotic cells
100
80
60
40
20
0
***
***
CON
RSC 1μM
RSC 3μM
5-FU 1μM
5-FU 3μM
MM-129 1μM
MM-129 3μM
b
HT-29
% of early and late apoptotic cells
50
25
0
***
***
CON
RSC 1μM
RSC 3μM
5-FU 1μM
5-FU 3μM
MM-129 1μM
MM-129 3μM
c
DLD-1
% of cells with decreased Δψm
80
60
40
20
0
***
***
CON
RSC 1μM
RSC 3μM
5-FU 1μM
5-FU 3μM
MM-129 1μM
MM-129 3μM
d
HT-29
% of cells with decreased Δψm
120
80
40
0
***
***
CON
RSC 1μM
RSC 3μM
5-FU 1μM
5-FU 3μM
MM-129 1μM
MM-129 3μM

Figure 2Sa

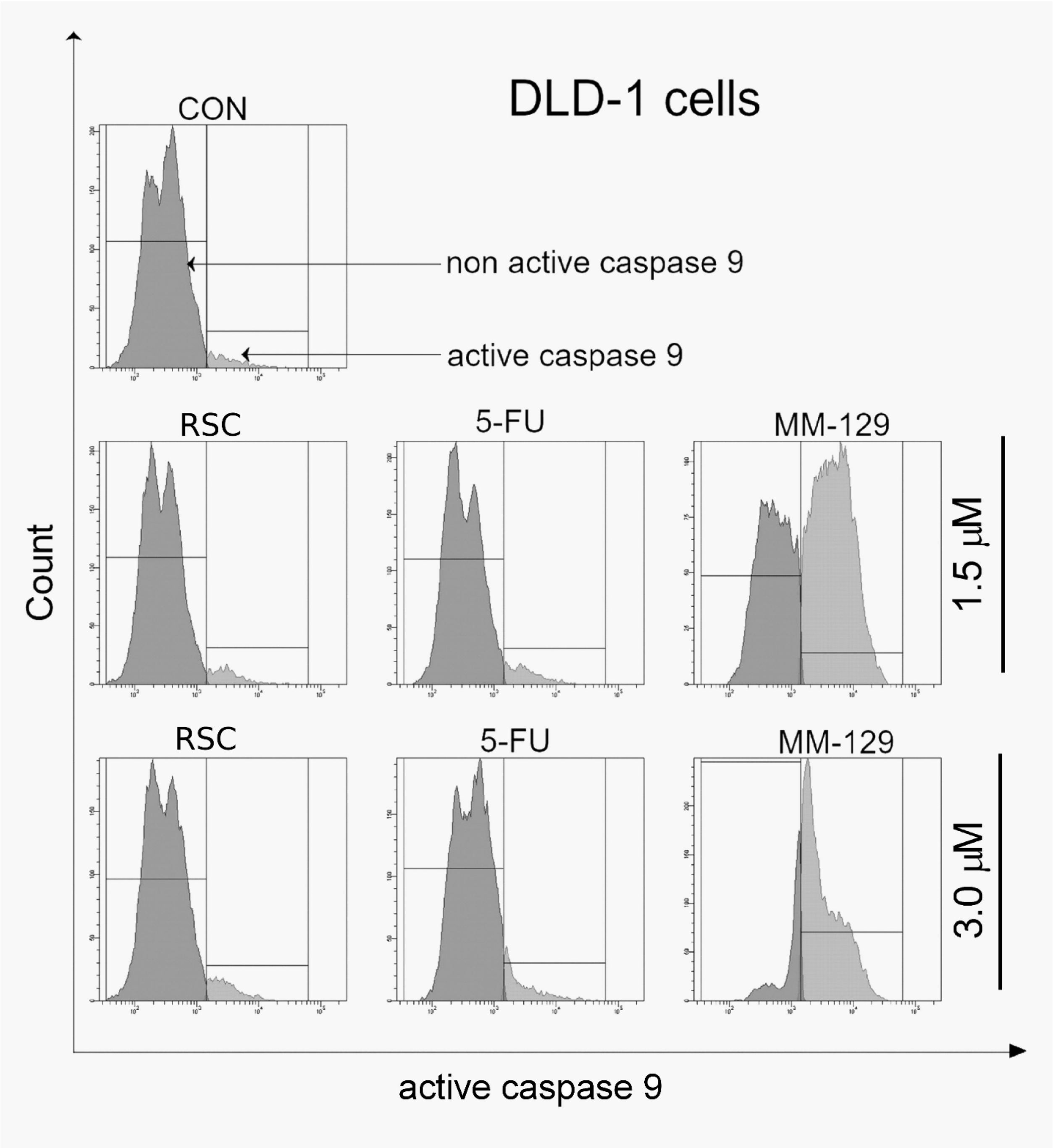
DLD-1 cells
CON
non active caspase 9
active caspase 9
RSC
5-FU
MM-129
1.5 µM
RSC
5-FU
MM-129
3.0 µM
Count
active caspase 9

Figure 2Sb

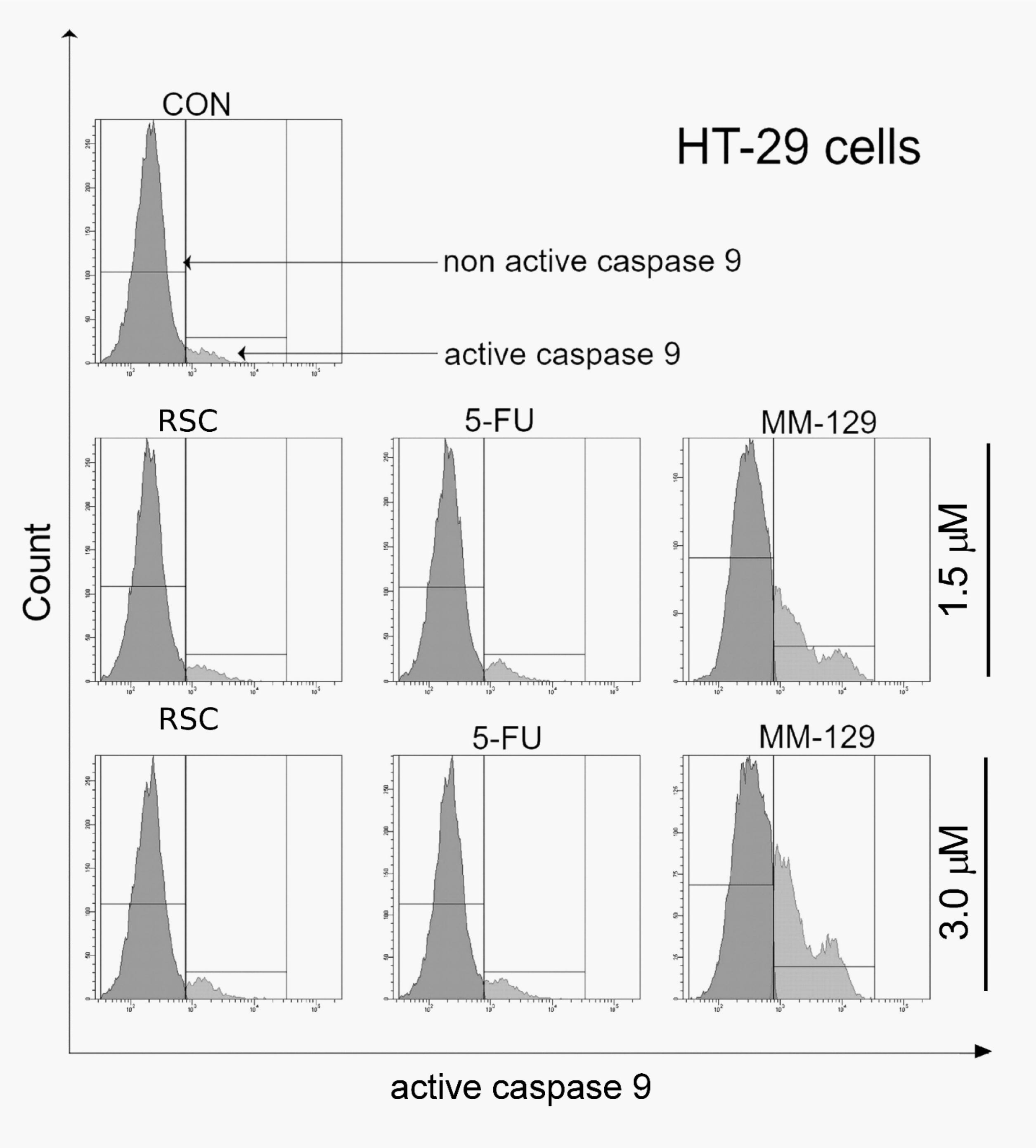
HT-29 cells
CON
non active caspase 9
active caspase 9
RSC
5-FU
MM-129
1.5 μM
RSC
5-FU
MM-129
3.0 μM
Count
active caspase 9

Figure 3Sa

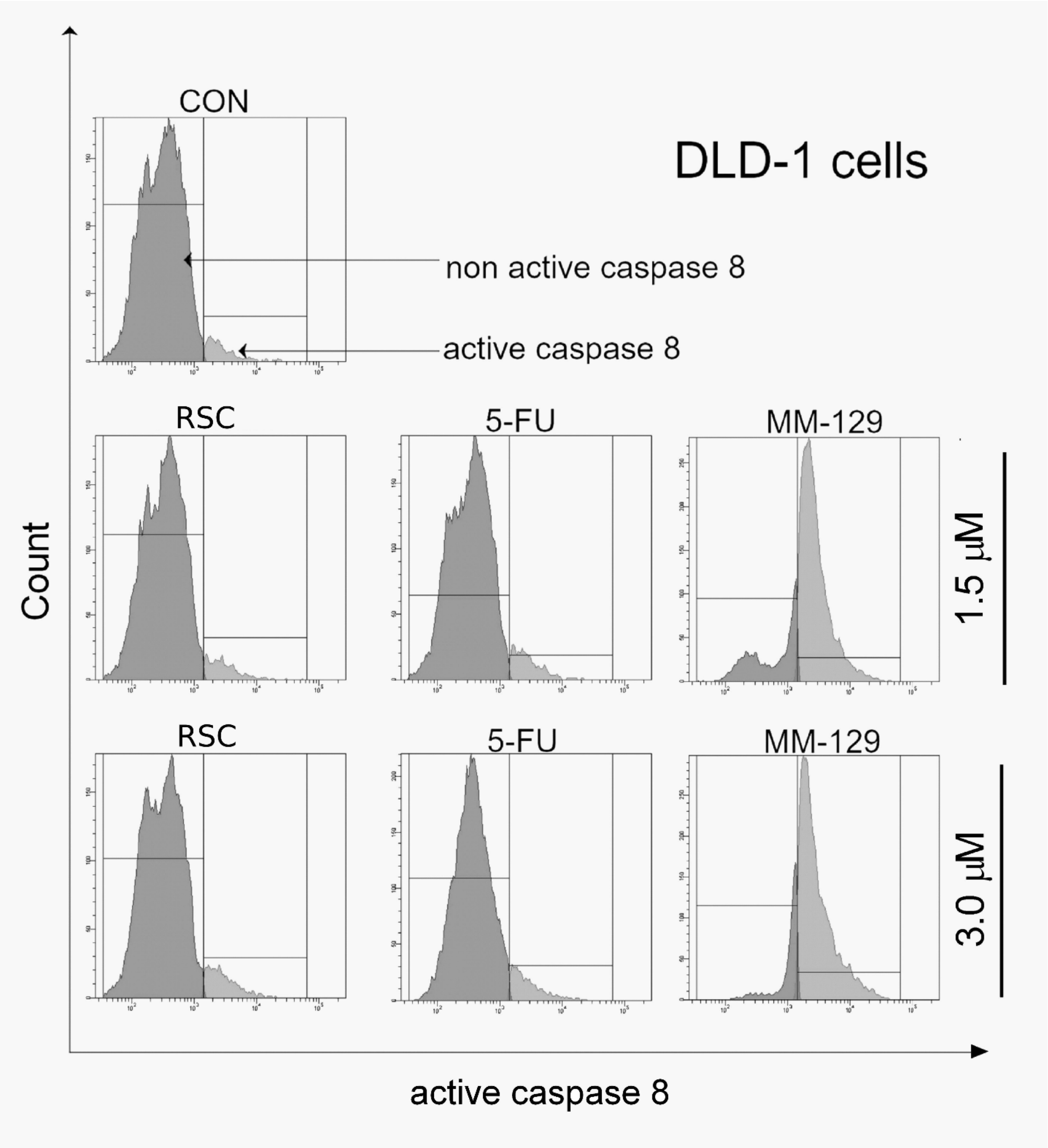

DLD-1 cells
CON
non active caspase 8
active caspase 8
RSC
5-FU
MM-129
1.5 μM
3.0 μM
Count
active caspase 8

Figure 3Sb

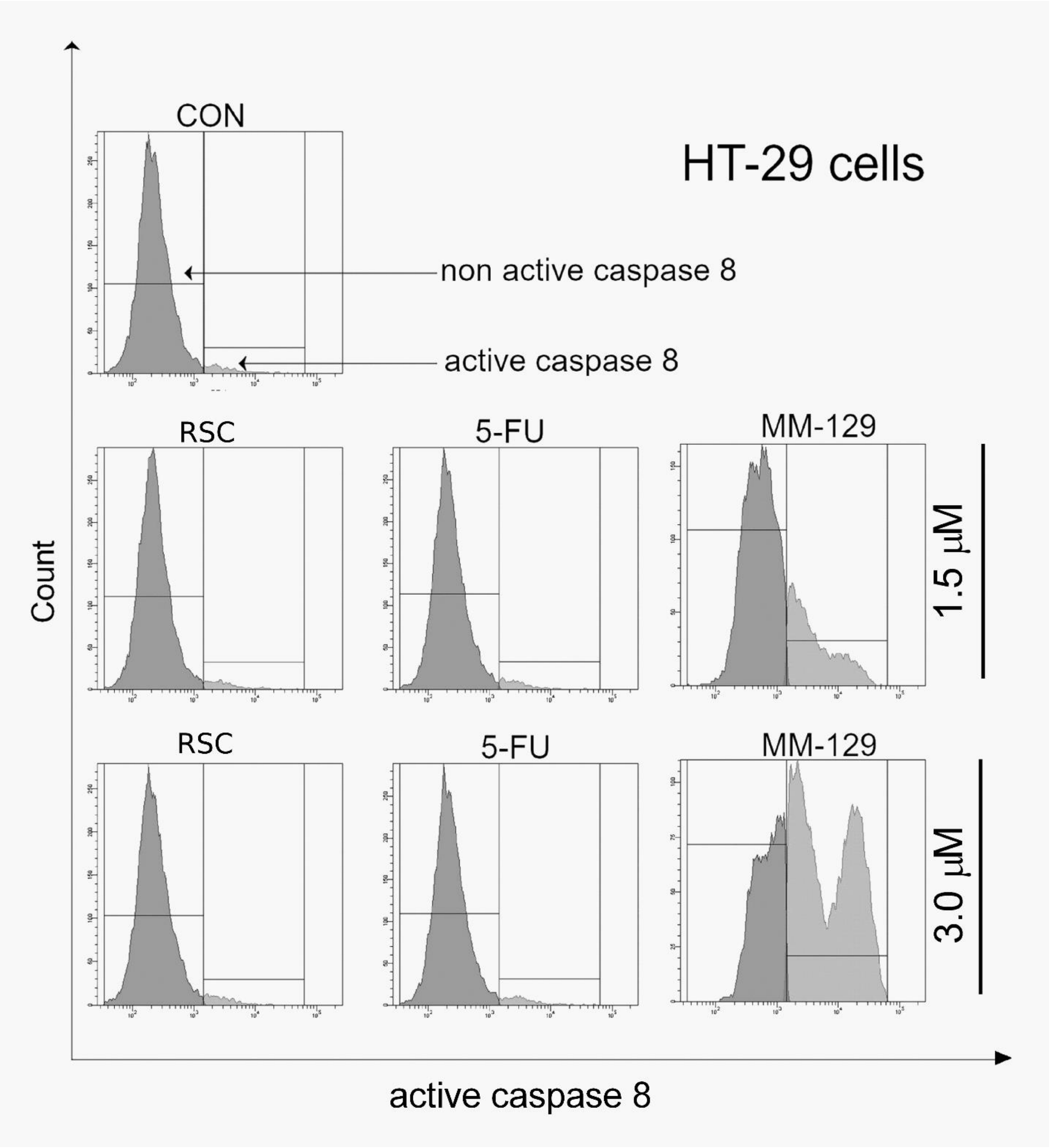
HT-29 cells
CON
non active caspase 8
active caspase 8
RSC
5-FU
MM-129
1.5 μM
RSC
5-FU
MM-129
3.0 μM
Count
active caspase 8

Figure 4Sa

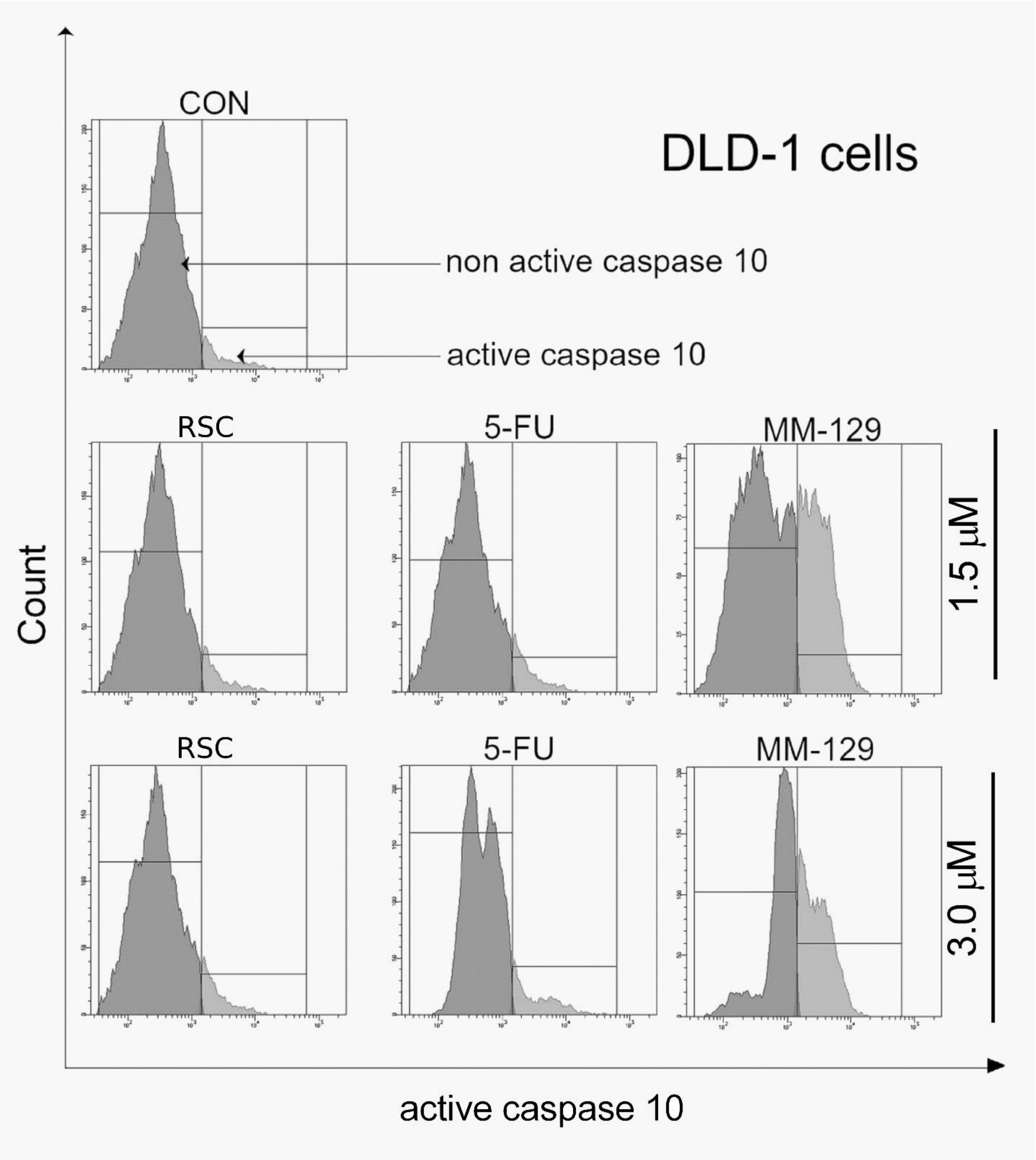

DLD-1 cells
CON
non active caspase 10
active caspase 10
RSC
5-FU
MM-129
1.5 μM
3.0 μM
Count
active caspase 10

Figure 4Sb

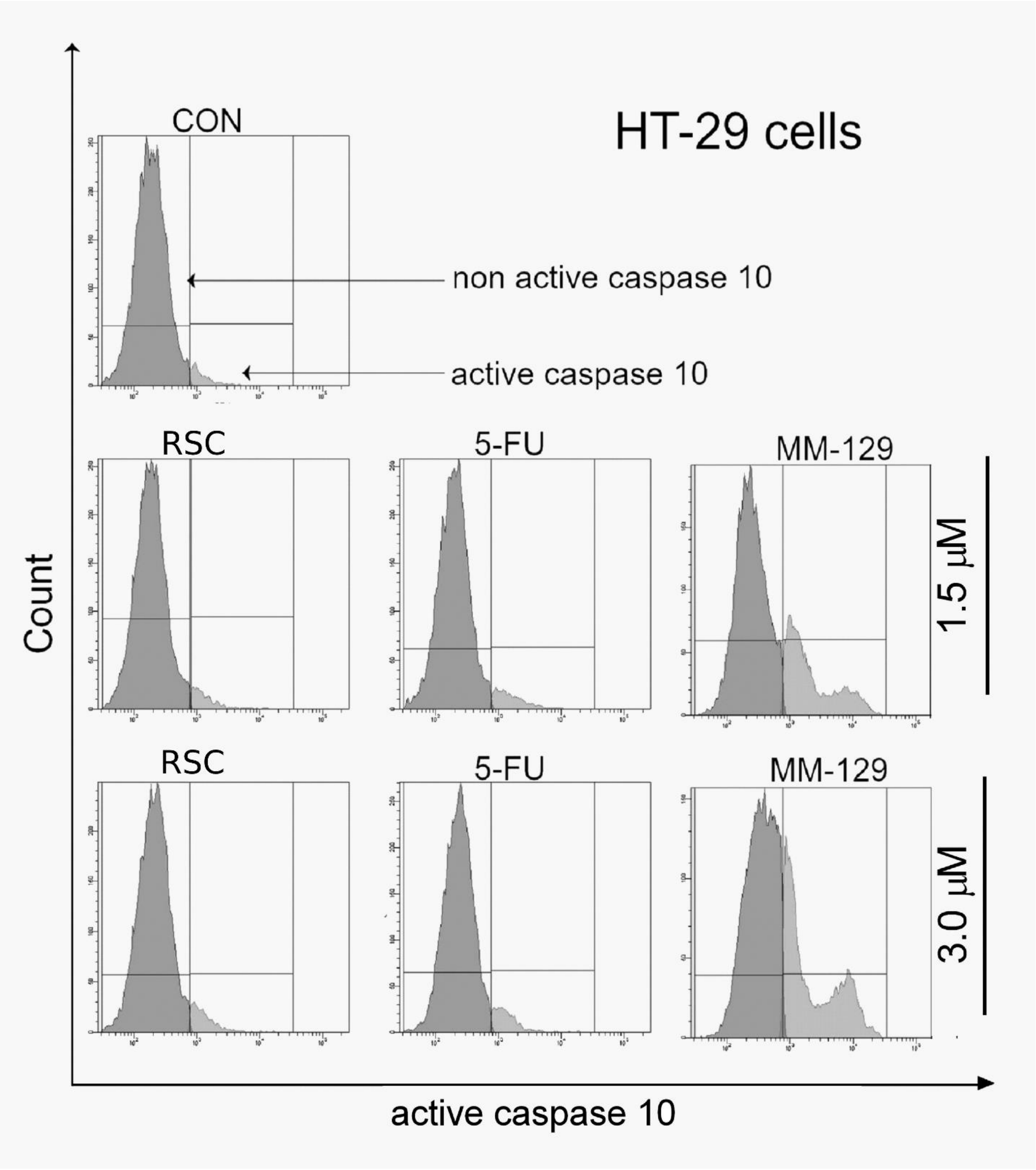

HT-29 cells
CON
non active caspase 10
active caspase 10
RSC
5-FU
MM-129
1.5 μM
RSC
5-FU
MM-129
3.0 μM
Count
active caspase 10

Figure 5Sa

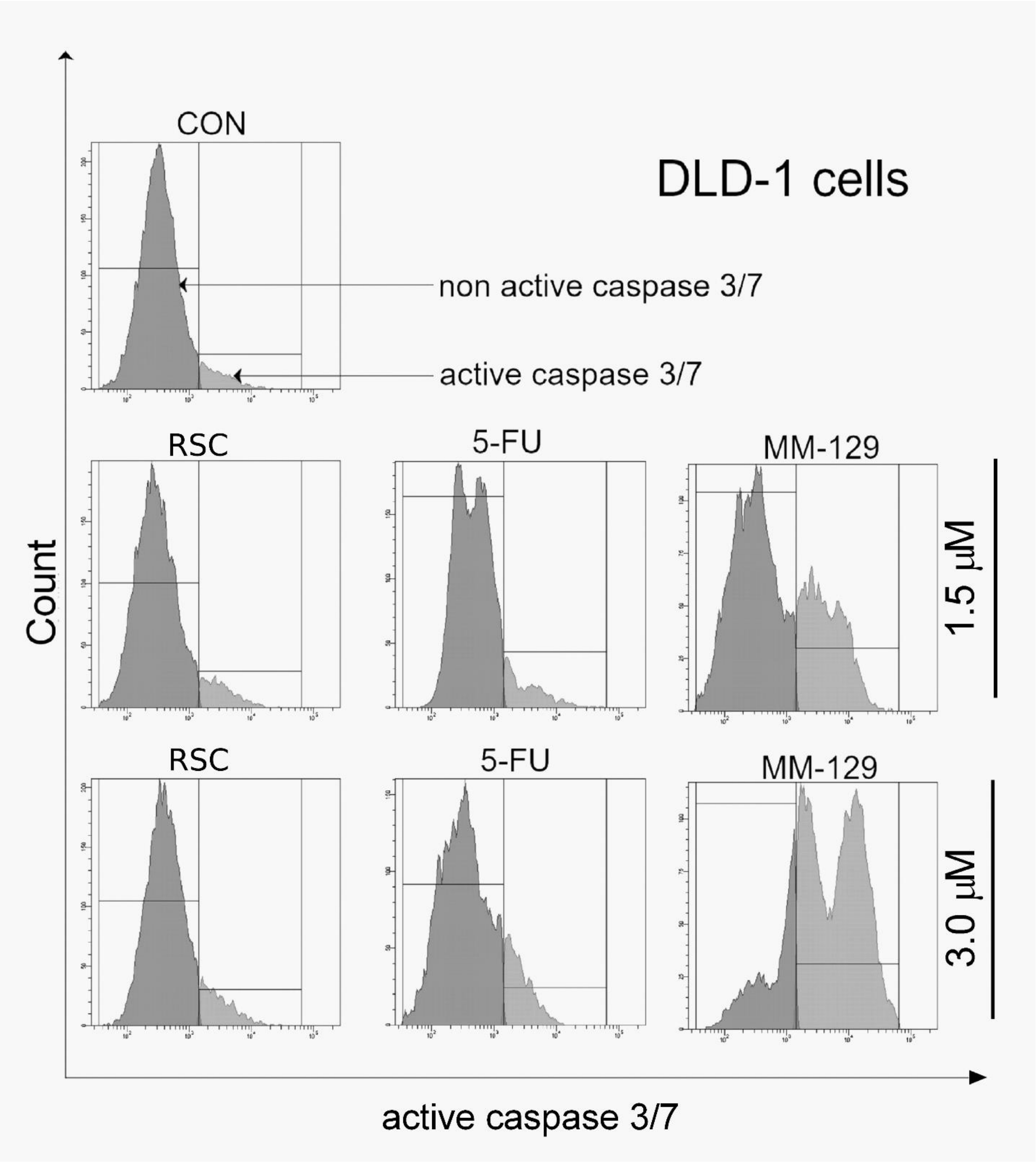
CON
DLD-1 cells
non active caspase 3/7
active caspase 3/7
RSC
5-FU
MM-129
1.5 μM
RSC
5-FU
MM-129
3.0 μM
Count
active caspase 3/7

Figure 5Sb

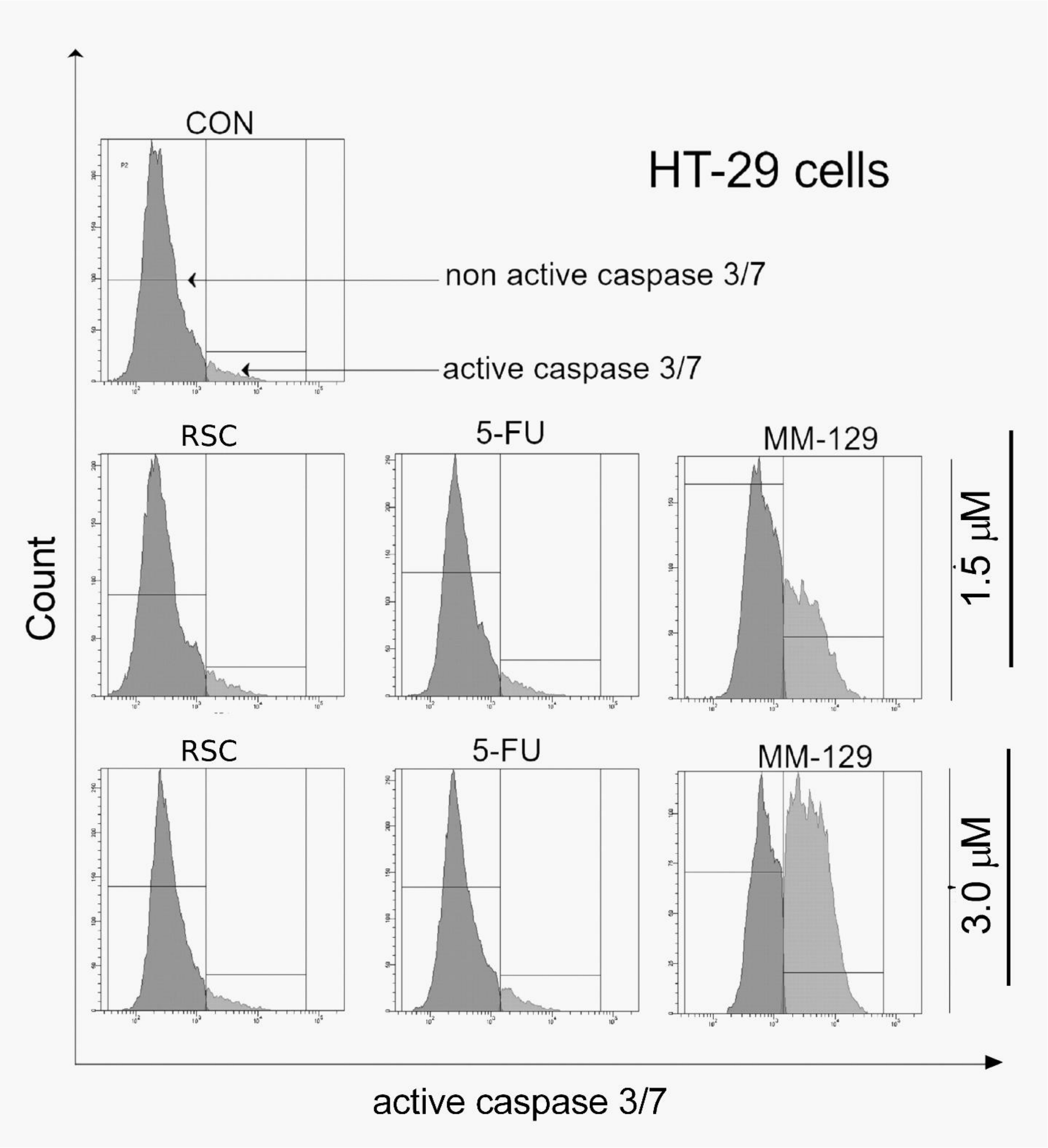
HT-29 cells
CON
non active caspase 3/7
active caspase 3/7
RSC
5-FU
MM-129
1.5 μM
3.0 μM
Count
active caspase 3/7

Figure 1S. Flow cytometric analysis of phosphatidylserine (PS) externalization. The percentage of early and late apoptotic cells of DLD-1 (a) and HT-29 (b) after 24 hours of incubation with roscovitine (RSC), 5-fluorouracil (5-FU) or MM-129 (1 μM and 3 μM). Flow cytometric analysis of mitochondrial membrane potential (ΔΨm) loss. The percentage of cells with decreased ΔΨm of DLD-1 (c) and HT-29 (d) treated for 24 hours with roscovitine (RSC), 5-fluorouracil (5-FU) and MM-129 (1 μM and 3 μM). Mean percentage values from three independent experiments (n=3) were presented as mean ± standard deviation (SD), and analyzed using one-way analysis of variance (ANOVA). ***$p<0.001$ vs. CON

Figure 2S. Representative flow cytometry dot-plots for caspase-9 activation in the populations of: DLD-1 (a) and HT-29 (b) colon cancer cells treated for 24 hours with roscovitine (RSC), 5-fluorouracil (5-FU) and MM-129 (1 μM and 3 μM).

Figure 3S. Representative flow cytometry dot-plots for caspase-8 activation in the populations of: DLD-1 (a) and HT-29 (b) colon cancer cells treated for 24 hours with roscovitine (RSC), 5-fluorouracil (5-FU) and MM-129 (1 μM and 3 μM).

Figure 4S. Representative flow cytometry dot-plots for caspase-10 activation in the populations of: DLD-1 (a) and HT-29 (b) colon cancer cells treated for 24 hours with roscovitine (RSC), 5-fluorouracil (5-FU) and MM-129 (1 μM and 3 μM).

Figure 5S. Representative flow cytometry dot-plots for caspase-3/7 activation in the populations of: DLD-1 (a) and HT-29 (b) colon cancer cells treated for 24 hours with roscovitine (RSC), 5-fluorouracil (5-FU) and MM-129 (1 μM and 3 μM).
